# Traumatic Events, Social Adversity and Discrimination as Risk Factors for Psychosis - An Umbrella Review

**DOI:** 10.3389/fpsyt.2021.665957

**Published:** 2021-10-22

**Authors:** Leonie Varchmin, Christiane Montag, Yvonne Treusch, Jakob Kaminski, Andreas Heinz

**Affiliations:** ^1^Department of Psychiatry and Psychotherapy, Charité Universitätsmedizin Berlin, Charité Campus Mitte (CCM), Berlin, Germany; ^2^Department of Psychiatry and Psychotherapy, University Clinic of the Charité in St. Hedwig Hospital, Charité Universitätsmedizin Berlin, Charité Campus Mitte (CCM), Berlin, Germany; ^3^Hochschule Döpfer (HSD) Döpfer, University of Applied Science, Cologne, Germany

**Keywords:** umbrella review, meta-analysis, discrimination, racism, migration, trauma, schizophrenia, psychosis

## Abstract

Exposure to childhood trauma is a well-known risk factor for severe mental disorders including schizophrenia and other non-affective psychoses. Beyond childhood trauma, there is increasing evidence that bullying, social exclusion, and discrimination during adolescence and adulthood may increase the risk of developing a psychotic disorder, and that such forms of traumatization may also underlie the elevated psychosis risk among migrants or persons with a visible minority status. In this umbrella review, we systematically assess meta-analyses regarding trauma and social adversity. A systematic literature review yielded 11 meta-analyses that met inclusion criteria and could be summarized quantitatively with a random effect model. Furthermore, six meta-analyses were evaluated qualitatively. Heterogeneity and publication bias were apparent in several meta-analyses. We observed that most significant social risk factors for psychosis were vulnerability for racist discrimination [OR = 3.90 (3.25–4.70)], migration [OR = 2.22 (1.75–2.80)], and childhood adversities [OR = 2.81 (2.03–3.83)]. Furthermore, social factors increasing the risk for psychosis were variation/impairment of parental communication, aversive adult life events, bullying, and factors associated with social isolation and discrimination. In spite of these environmental risk factors, there is a lack of evidence regarding treatment of trauma and psychosis, although some psychotherapeutic and art therapy approaches appear to be promising. Beyond individual interventions, stigmatization, racism, and other forms of discrimination need to be targeted to increase solidarity and communal support.

## Introduction

Schizophrenia is a serious mental disorder characterized by altered experience of the environment including hallucinations, self-disorders, delusions, and negative symptoms (1, 2). The WHO study suggested rather similar incidence rates worldwide, with incidence ranging between 0.1 and 0.4 per 1,000 individuals per year (3). The rather uniform psychosis risk in several countries and cultures may suggest that schizophrenia is a ubiquitous phenomenon, inherited in human nature, and indeed, a substantial genetic contribution to psychosis risk was confirmed (4). On the other hand, environmental risk factors play a significant role, as evinced by the strong increase in psychosis risk among certain populations of first- and second-generation migrants and refugees (5–9). Increased psychosis risk among migrants and refugees is not simply explained by genetic factors, as there is no evidence for increased schizophrenia rates in the countries of origin (10). Instead, it has been observed that a low density of persons with a visible minority status in the neighborhood is associated with increased schizophrenia risk (6), suggesting that lack of social support and (racist or other forms of) discrimination contribute to psychosis risk (6, 8). As refugees display even higher rates of psychotic experiences than migrants without a refugee status (9), traumatization due to war experiences or during dangerous escapes and travels may contribute to vulnerability. In a recent umbrella review by Radua et al. (11) examining the strength of evidence for risk and protective factors (socio-demographic, parental, perinatal, later factors, or antecedents) for psychosis, strong evidence was found for ultra-high risk states [a state operationalized with varying diagnostic tools (12), in which psychotic experiences occur, however, not (yet) to the extent of a full blown psychotic episode] and for ethnic minority status, i.e., belonging to the so-called Black-Caribbean ethnicity in England.

Other forms of traumatization and stress exposure have also been implicated in the development of psychotic experiences. A series of studies show that childhood abuse is a prominent risk factor (13–18). In recent years, research related to those factors shifted its focus toward an approach that distinguishes between different types of childhood abuse (e.g., sexual vs. physical, emotional abuse, or neglect) and that considers the effects of trauma on specific psychotic experiences and their severity (16, 17). A meta-analysis of retrospective studies found prevalence rates of childhood sexual abuse of 26.3% (21.2–32.2), of childhood physical abuse of 38.8% (36.2–42.4), and of childhood emotional abuse of 34% (29.7–38.5) in patients with psychosis (19), highlighting the relevance of the possible link between trauma and psychosis. Stressful and potentially traumatizing experiences contributing to childhood adversity, furthermore, include bullying (18, 20), parental death (21), and alterations in parental communication (22, 23). Also, traumatic experiences during adulthood can contribute to psychosis risk (24), which may be explained by an explanatory framework that incorporates computational models on how our mind reacts on changing and potentially threatening environments including social exclusion and experiences of discrimination (8). In this context, a Bayesian framework suggests that prior knowledge about the world is always compared with sensory input; the difference between the estimated likelihood of an event (e.g., sensory input) and prior beliefs concerning such an event (expectation), each weighted by a certain precision, results in a so-called prediction error, which serves to update priors (25). In case of imprecise prior knowledge, prediction errors increase; as far as they are encoded by mesolimbic dopamine, elevated phasic dopamine release can increase the signal-to-noise ratio, although at the price of attributing salience to otherwise irrelevant stimuli, thus, linking a Bayesian account with dopamine dysfunction in schizophrenia (26, 27). We suggest that there are several reasons why prior knowledge may be challenged, thus, resulting in imprecise neurobiological encoding of priors (8). Specifically, imprecise encoding of prior knowledge may not only result from predominantly biological causes [e.g., anti-NMDA receptor antibodies in some psychotic states (28)], but also arise in complex situations characterized by threatening experiences and potentially uncontrollable social interactions as, e.g., experienced by previously traumatized or ethnically discriminated individuals (8, 29). Previous studies found varying prevalence rates between 0 and 55% of PTSD in patients suffering from schizophrenia spectrum disorders (30), suggesting a rather high prevalence of PTSD which may often remain overlooked in clinical settings (31). Further studies focused on the impact of urbanicity and poverty (32–35), poor medical care [particularly obstetric complications (36)], and drug use, particularly cannabis (37, 38).

In our umbrella review, we systematically research and summarize meta-analyses regarding trauma and related risk factors as identified by database screening and subsequent key word identification. We hypothesized that traumatic events in childhood and adulthood can trigger psychotic experiences (26), systematically reviewed the literature, focused on potentially traumatic experiences addressed in at least two previously published meta-analyses, and preregistered our respective hypotheses. We found three factors that fulfilled inclusion criteria and that were related to trauma, discrimination, and social adversity, migration, vulnerability for ethnic discrimination, and childhood trauma. We hypothesized to find variable heterogeneity depending on the examined factors. Our work thus extends a previous umbrella review by Radua et al. (11) by providing meta-analyses on three socially highly relevant and empirically well-replicated risk factors (discrimination, migration, and childhood traumatization), thus allowing a quantitative estimation of effect sizes and heterogeneity. Our umbrella review also includes more recently published meta-analyses on refugee status and psychosis (9), as well as migration and psychosis (8, 39). Where possible, we present a calculation of common effect sizes for a direct visualization of the heterogeneity. We address controversies regarding specific associations and discuss evidence regarding therapeutic interventions.

## Methods

For this study, we followed the Preferred Reporting Items for Systematic Reviews and Meta-Analyses (PRISMA) guideline (40). Methods of the analysis and inclusion criteria were specified in advance, documented, and pre-registered (41). Additionally, we followed the guideline by Fusar-Poli and Radua (42) that provides instructions for the production of umbrella reviews. For the purpose of this study, we refer to the definition of trauma from the ICD-10, where post-traumatic stress disorders arise “as a delayed response to a stressful event or situation (of either brief or long) duration of an exceptionally threatening or catastrophic nature, which is likely to cause pervasive distress in almost anyone” (2). Accordingly, childhood trauma is a form of trauma that appears before the 18th birthday, that results from either emotional, physical, sexual abuse, or neglect, and that can be assessed by common interview measures such as the childhood trauma questionnaire (43). Childhood adversities cover childhood trauma as well as other forms of potentially traumatizing events in childhood such as bullying, parental death, and alterations in parental communication. All risk factors examined are social environmental risk factors (in contrast to physical environmental risk factors) (44).

### Eligibility Criteria

#### Types of Studies

We searched meta-analyses assessing trauma and related risk factors associated with the incidence of non-affective psychosis in different subgroups. Search terms were chosen on the basis of a first screening of the PubMed database with a restriction of year of publication between January 2018 and December 2020 using the following search terms on August 13, 2020:

Trauma psychosis

The screening yielded 513 original research articles that were all assessed *via* their abstract by author L.V. Among these articles, 59 original studies assessed the association of trauma and related risk factors for psychosis. The authors LV, JK, and AH identified trauma, racism, discrimination, and migration as the most relevant keywords regarding trauma and social adversity as environmental risk factors on the basis of mutual agreement. For the purpose of this study, keywords regarding genetic risk factors, other environmental non-traumatic risk factors (e.g., infections), or drug abuse were not regarded eligible as potential keywords.

The keyword list served as search terms for the second systematic search on PubMed conducted by L.V. No limits for language or publication date were applied, and unpublished material was excluded. The search was run on August 31, 2020. The date for the literature search reported in the preregistration was mistakenly stated to be August 31st, 2019. Please note that 2020 is the correct year. This systematic search applied the following search terms:

(Trauma OR Migration OR Discrimination OR Racism) AND (Psychosis OR non-affective psychosis OR schizophrenia OR first episode psychosis) AND meta-analysis

This database search yielded 139 records without duplicates, which were all screened *via* their abstract by LV. For the purpose of this study, we only selected meta-analyses (*n* = 18). According to a request of a reviewer, we performed a complementary database search based on the same search terms on Embase, PsychInfo, and Web of Science (restricted to results published until August 31, 2020 in accordance with our preregistered search).

#### Inclusion Criteria

In order to be considered for the meta-analysis, studies were required to (i) report a pooled risk ratio (RR, IRR, HR, or OR) with a 95% confidence interval; or (ii) an effect size that was presented in a way that could be converted to the common effect size of Cohen's d (e.g., Pearson's correlation coefficient r) of the incidence of positive or negative symptoms or diagnosed schizophrenia (SCZ), other non-affective psychotic disorders (NAPs), or first episode psychosis (FEP) according to standard operationalized criteria. All studies had to assess (iii) a risk factor described above (i.e., trauma, or related social adversity, or a history of migration, or minority status). Finally, all studies must have had a reference population (iv), and must have been published in a peer reviewed journal (v).

#### Exclusion Criteria

Studies were excluded when (i) the patient group involved individuals with a drug-related-psychosis, (ii) the pooled effect size was presented in a way that was not convertible to a common effect size, (iii) the article turned out to present original data only without a calculation of pooled effect sizes, thus, rather representing a systematic review instead of a meta-analysis. In addition (iv), and for reasons of parsimony, we also did not include meta-analyses and reviews that solely focus on the country of origin or destination of migration.

#### Quality Assessment

To the best of our knowledge, there are no consented measurement tools or guidelines for evaluating the quality of meta-analyses included in an umbrella review. Therefore, we adapted the AMSTAR- instrument established by Shea et al. (45), which was originally designed for assessing the methodological quality of systematic reviews.

#### Data Extraction Process

LV extracted the data and JK and AH checked the extracted data. Disagreements were resolved by discussion between the authors. Samples of the original meta-analyses used in this article had to be independent to ensure trustworthy results for a new pooled summary effect size. However, in several analyses, there was an overlap of original studies included into several meta-analyses that accounted for the same factor. In this case, the summary effect size calculated with more studies was preferred, while the other effect size was excluded.

#### Data Items

All pooled effect sizes and their confidence interval reported in the meta-analyses were recorded in the (Supplementary Tables 1–3), which includes information about the examined factor, possible adjustments (e.g., age, gender/sex, socioeconomic status, the diagnostic inclusion criteria [e.g., SCZ, NAP, FEP, psychotic disorder (PD)]), the number of studies (k) included for the calculation of the pooled effect, the number of cases (n1), controls (n2) and the *p*-value. The effect size values were converted to the common effect size “Cohen's d” as described below, and then also listed. If available, measures of heterogeneity such as Cochran's Q (46) and *I*^2^- statistics (47) were reported. Additionally, we recorded indications for publication bias. If the information about publication bias was reported, the method for its estimate including visual inspection of the funnel plot, Egger's test (48), the Fail-Safe N test (49), the trim-and-fill-method (50), or the LFK index (51) are included.

#### Review and Meta-Analysis

The individual meta-analyses were grouped by similarity of factors or subgroups examined, respectively. The following factors could be identified as potential candidates for the calculation of a pooled common effect size: (I) psychosis and childhood adversity—(Ia) childhood trauma (sexual abuse, physical abuse, emotional/psychological abuse, neglect), (Ib) bullying in childhood, (Ic) parental death, (Id) variations in parental communication, (Ie) psychosis and aversive adult life events (Table 1); (II) psychosis and migration—(IIa) first generation migrants vs. second generation migrants, (IIb) refugee status (Table 2), (IIIc) age at migration; (III) vulnerability for ethnic discrimination [proxied by minority status/skin-color (IIIa) and ethnic density effects (IIIb) (Table 3)]; (IV) psychosis and urbanicity (Table 4); (V) psychosis and obstetric complications. The calculation of a new summary common effect size was possible if (viii) more than one meta-analyses existed, and (ix) if the existing pooled effect sizes were convertible to Cohen's d. If the calculation of a common effect size was not possible, the factor was still qualitatively reviewed as for Id, Ie, IIb, IV, V. In addition to pooling of the factors mentioned above, one meta-analysis (VI) was conducted to assess moderating effects and compare the summary effect sizes of childhood-adversities (proxied by total childhood trauma) with those of migration (proxied by first and second generation migration) and minority status/vulnerability for ethnic discrimination (proxied by black skin color). We grouped social risk factors potentially associated with trauma and reported in the original meta-analyses into (1) childhood trauma, (2) migration, and (3) visible minority status that may increase vulnerability for racist discrimination. These groupings and labels represent our own classification based on previous meta-analysis and conceptual reviews (8, 26, 29, 30), and are based on the preregistered literature review with the above-mentioned inclusion and exclusion criteria, aiming at a fine-grained evaluation of social adversity.

**Table 1:** Childhood trauma, other childhood adversities, adult life events, and psychosis risk.

**Factor/study**	**Diagnosis**	**k1**	**k2**	**Summary statistical value^**+**^ or common size effect^**++**^ and variance**	***I* ^**2**^**	***Q***
**Childhood trauma**						
Total	PD, PS	2		***d*** **=** **0.57 (0.39–0.74)**	0	0.11^ns^
**Specific trauma type**						
Sexual abuse	SCZ, Dis, PD, PS	2		***d*** **=** **0.50 (0.39–0.62)**	10.0	1.11*
Physical abuse	SCZ, Dis, PD, PS	2		***d*** **=** **0.63 (0.51–0.74)**	0	0.49 ^ns^
Emotional abuse	SCZ, Dis, PD, PS	2		***d*** **=** **0.77 (0.53–1.01)**	11.6	1.13*
Neglect	SCZ, Dis, PD, PS	2		***d*** **=** **0.47 (0.34–0.60)**	0	0.76 ^ns^
**Other childhood adversities**						
Bullying in childhood	PD, PS	2		***d*** **=** **0.49 (0.37**–**0.62)**	0	0.16 ^ns^
Parental death	PD, PS	2		***d*** **=** **0.12 (0.04**–**0.21)**	0	0.71 ^ns^
Variations in parental communication De Sousa et al.	PD		19	*d* = 0.97 (0.76–1.18)	46.5	33.4****
**Adult life events**						
Beards et al.	PD, PE		13	*D* = 0.64 (0.42–0.86)	87.3	

Summary effects and qualitative review of included 1meta-analyses. PD, psychotic disorder; SCZ, schizophrenia; PE, psychotic experiences; PS, psychotic symptoms; Dis, dissociation; k1, number of meta-analysis for summary effect size; k2, number of effect sizes included in study; d, Cohen's d; ^+^pooled result of several meta-analyses written in bold letters; ^++^one meta-analysis available, result converted in common effect size and presented qualitatively; Q, Cochran's Q heterogeneity statistics; I^2^, I^2^ index for heterogeneity. *p < 0.3; **p < 0.05; ***p < 0.01; ****p < 0.001; ^ns^, not significant.

**Table 2:** First- and second-generation migrants, refugee status, and psychosis risk.

**Study**	**Diagnosis**	**K**	**n1**	**n2**	**Statistical value and variance**	**Summary statistical value^**+**^ or common size effect^**++**^ and variance**	***I* ^**2**^**	**Q**
**First- and second-generation migrants, high-quality studies**								
Selten et al.	NAP	15	4,896	18,040	RR = 2.15 (1.95–2.37)*	*d* = 0.42 (0.37–0.48)	94.7	
Henssler et al.	NAP	25			RR = 1.81 (1.62–2.02)	*d* = 0.33 (0.27 −0.39)	97.6	
Cantor-Gr et al.	SCZ	50	3,092	27,130	RR = 2.90 (2.50–3.40)	*d* = 0.59 (0.51–0.68)		68.3**
**Total**						***d*** **=** **0.44 (0.11–0.77)**	91.8	24.5***
**First-generation migrants**								
Selten et al.	PD	29	14,351	84,701	RR = 2.55 (2.31–2.82)	*d* = 0.52 (0.46–0.57)	97.9	
Henssler et al.	NAP	20			RR = 1.81 (1.59–2.07)	*d* = 0.33 (0.26–0.40)	97.6	
Cantor-Gr. et al.	SCZ	40	2,846	26,785	RR = 2.7 (2.3–3.2)	*d* = 0.55 (0.46–0.64)		55.4**
Bourque et al.	PD	61	5,556	33,160	IRR = 2.3 (2.0–2.7)	*d* = 0.46 (0.38–0.55)	94.4	1071.0***
**Total**						***d*** **=** **0.46 (0.37**–**0.56)**	85.2	20.3***
**Second-generation migrants**								
Selten et al.	PD	13			RR = 1.78 (1.66–1.90)***	*d* = 0.32 (0.28–0.35)	94.2	
Henssler et al.	NAP	13			RR = 1.82 (1.66–1.99)	*d* = 0.33 (0.28–0.38)	90.5	
Cantor-Gr. et al.	SCZ	7	474	8,895	RR = 4.5 (1.5–13.1)	*d* = 0.82 (0.22–1.42)	4.5	55.4
Bourque et al.	PD	28	4,515	24,360	IRR = 2.1 (1.8–2.5)	*d* = 0.41 (0.32–0.51)	91.1	303.0***
**Total**						***d*** **=** **0.34 (0.29–0.40)**	53.4	6.4*
**Refugees**								
Brandt et al.	NAP	10			RR = 2.52 (1.78–3.57)****	*d* = 0.51 (0.32–0.70)	98.0	
Selten et al.	NAP	4			RR = 1.88 (1.57–2.24)	*D* = 0.35 (0.25–0.45)	91.4	
**Total**						***D*** **=** **0.41 (0.25–0.56)**	54.6	2.20*

Summary effects and qualitative review of included meta-analyses. PD, psychotic disorder; NAP, non-affective psychosis; SCZ, schizophrenia. K, number of effect sizes; n1, number of cases; n2, number of controls RR, risk ratio; IRR, incidence rate ratio; d, Cohen's d; ^+^pooled result of several meta-analyses written in bold letters; ^++^one meta-analysis available, result converted in common effect size and presented qualitatively; Q, Cochran's Q heterogeneity statistics; I^2^, I^2^ index for heterogeneity. *p < 0.3; **p < 0.05; ***p < 0.01; ****p < 0.001.

**Table 3:** Vulnerability for racist discrimination and psychosis risk.

**Study**	**F/S**	**Dia-gnosis**	**k**	**n1**	**n2**	**Statistical value and variance**	**Summary statistical value^**+**^ or common size effect^**++**^ and variance**	***I* ^**2**^**	**Q**
**Skin color white**									
Selten et al.	F + S	NAP	19			RR = 1.65 (1.46–1.85)	*d* = 0.27 (0.21–0.34)	97.1	
Cantor-Gr. et al.	F + S	SCZ	16	799	1,5902	RR = 2.3 (1.8–3.0)	*d* = 0.46 (0.32–0.61)		
Borque et al.	F	PD	19	1,808	20,853	IRR = 1.8 (1.6–2.1)	*d* = 0.33 (0.26–0.41)	89.7	175.4***
Borque et al.	F	PD	4	243	5,566	IRR = 1.9 (1.2–3.0)	*d* = 0.35 (0.10–0.61)	87.2	23.5***
**Total**							***d*** **=** **0.34 (0.26**–**0.41)**	51.5	6.18*
**Skin color black**									
Selten et al.	F + S	NAP	23			RR = 4.19 (3.42–5.14)****	*d* = 0.79 (0.68–0.90)	94.3	
Cantor-Gr. et al.	F + S	SCZ	16	896	24,931	RR = 4.8 (3.7–6.2)	*d* = 0.86 (0.72–1.01)		
Olbert et al.	BI	SCZ	52	863,293	2,532,655	OR = 2.42 (1.59–3.66)****	*d* = 0.49 (0.25–0.71)	98.3	
Borque et al.	F	PD	18	1,711	25,255	IRR = 4.0 (3.4–4.6)	*d* = 0.76 (0.67–0.84)	79	80.8***
Borque et al.	F	PD	7	127	279	IRR = 5.4 (3.2–8.8)	*d* = 0.92 (0.64–1.19)	78.9	28.4***
**Total**							***d*** **=** **0.77 (0.67–0.87)**	54.8	8.85*
**Skin color other**									
Selten et al.	F + S	NAP	11			RR = 1.73 (1.41–2.14)	*d* = 0.30 (0.18–0.42)	95.1	
Cantor-Gr. et al.	F + S	SCZ	11	649	13,782	RR = 2.2 (1.6–3.0)	*d* = 0.43 (0.26–0.61)		
Borque et al.	F	PD	16	505	14,765	IRR = 2.0 (1.6–2.5)	*d* = 0.38 (0.26–0.51)	84.7	97.8***
Borque et al.	F	PD	5	51	8,843	IRR = 2.0 (1.0–4.0)	*d* = 0.38 (0.00–0.76)	73.8	15.3***
**Total**							***d*** **=** **0.36 (0.28**–**0.43)**	0	1.7^ns^
**Ethnic density**									
**High**									
Bosqoui et al.		PD	5			IRR = 2.52 (1.28–5.32)	*d* = 0.51 (0.14–0.92)	0	
**Low**									
Bosqoui et al.		PD	5			IRR = 4.51 (2.25–8.58)	*d* = 0.83 (0.45–1.19)	0	

Summary effects and qualitative review of included meta-analyses. F, first-generation migrant; S, second-generation migrant; BI, black individuals; PD, psychotic disorder; NAP, non-affective psychosis. SCZ, schizophrenia; k, number of effect sizes; n1, number of cases; n2, number of controls; OR, odds ratio; RR, risk ratio; IRR, incidence rate ratio; d, Cohen's d; ^+^pooled result of several meta-analyses written in bold letters; ^++^one meta-analysis available, result converted in common effect size and presented qualitatively; Q, Cochran's Q heterogeneity statistics; I^2^, I^2^ index for heterogeneity. *p < 0.3; **p < 0.05; ***p < 0.01;****p < 0.001; ^ns^, not significant.

**Table 4:** Urbanicity and psychosis risk.

**Study**	**Dia-gnosis**	**k**	**n1**	**n2**	**Statistical value and variance**	**Summary statistical value^**+**^ or common size effect^**++**^ and variance**	***I* ^**2**^**	***Q***
**Urbanicity**								
Kirkbride et al.	NAP	9			IRR = 1.02 (1.02–1.03)***	*d* = 0.01 (0.01–0.02)		
Kirkbride et al.	SCZ	15			IRR = 1.03 (1.01–1.03)***	*d* = 0.02 (0.01–0.02)		
Castillejos et el.	NAP	5			IRR = 2.25 (2.00–2.52)****	*d* = 0.45 (0.38–0.51)		
Castillejos et el.	SCZ	3			IRR = 1.64 (1.38–1.95)***	*d* = 0.27 (0.18−0.37)		
**Total**						***d*** **=** **0.57 (0.39−0.74)**	98.5	206.71***

Summary effects and qualitative review of included meta-analyses. NAP, non-affective psychosis; SCZ, schizophrenia; k, number of effect sizes; n1, number of cases; n2, number of controls; IRR, incidence rate ratio; d, Cohen's d; ^**+**^pooled result of several meta-analyses written in bold letters; ^++^one meta-analysis available, result converted in common effect size and presented qualitatively; Q, Cochran's Q heterogeneity statistics; I^2^, I^2^ index for heterogeneity. *p < 0.3; **p < 0.05; ***p < 0.01; ****p < 0.001; ^ns^, not significant.

#### Summary Measures

We followed the formulas provided by Fusar-Poli et al. (42), where the risk ratio (RR) can be obtained as a function of incidence rate ratio (IRR):


$$\begin{array}{l} RR=\frac{average\;\left(time_{exposed}\right)}{average\;\left(time_{non-exposed}\right)}\times IRR \end{array}$$

As incidences are small (42):


$$\begin{array}{l} RR\;\approx IRR \end{array}$$

The odds ratio (OR) can be obtained as a function of the risk ratio:


$$\begin{array}{l} OR=\frac{1-p_{non-exposed}}{1-p_{exposed}}\;\times RR \end{array}$$

As probabilities of developing the disease (p) are small (42):


$$\begin{array}{l} OR\;\approx RR \end{array}$$

Hence, we could assume as far as incidences are not too large that:


$$\begin{array}{l} OR\;\approx RR\;\approx IRR \end{array}$$

We converted OR, RR, and IRR to common effect size Cohen's d using the formula provided by Borenstein et al. (52):


$$\begin{array}{l} d=\frac{\ln\left(OR;RR;IRR\right)\times \sqrt{3}}{\pi } \end{array}$$

In case authors presented their results with the Pearson's correlation coefficient r, the conversion was possible with the help of the formula provided by Fusar-Poli et al. (42):


$$\begin{array}{l} d=\frac{2r}{\sqrt{1-r^{2}}} \end{array}$$

In case authors presented their results with the help of the Hedge's g measure, we used the approximation by Fusar-Poli et al. (42), where for sample sizes that are large enough:


$$\begin{array}{l} d\;\approx g \end{array}$$

The common effect sizes could now be used to calculate a summary effect size for factors examined by more than one study. Finally, summary effect sizes could be reconverted to odds ratios to facilitate interpretation:


$$\begin{array}{l} OR=e^{\frac{d*\pi }{\sqrt{3}}} \end{array}$$

We used Harrer et al. (53) for the calculations of our summary effect sizes and the creation of our forest plot with the help of the statistics software RStudio (54). In detail, we used packages “tidyverse,” “meta,” “metafor,” and “dmetar.” We pooled effect sizes using a random effects model included in the “metagen”-function. Random effect models are preferred for studies consisting of differing populations (55) and therefore account also for the error resulting from distributional effects of true size effects. The function “metagen,” applies the inverse variance method for weighing (56) and uses the “DerSimonian-Laird”-method (57) to obtain the between-study-variance estimator for τ^2^, and the Jackson method for confidence interval of τ^2^. The measurement of the output value is the standardized mean difference (SMD), which is identical to Cohen's d (58). Forest plots were generated with the function “meta::forest.” The script and the excel sheet required to run it can be found on Github (see data availability statement).

We used Cohen's d, which facilitates the comparison with the effects of different studies independent of the original way of their measurements (59). A commonly used interpretation categorizes effect sizes |d| <0.2 as small, |d| <0.5 as medium and |d| <0.8 as large (60).

#### Heterogeneity

Heterogeneity was assessed using Q statistics (46). The computation of the *I*^2^ -index (47) represents the percentage of variance caused by heterogeneity (61): *I*^2^ values close to 0% indicate that heterogeneity is primarily due to sampling error within the studies, *I*^2^ values <25% represent low, <50% moderate, <75% high, and >75% substantial heterogeneity due to between-study variability (e.g., method used, sample population) (47).

#### Biases

The possibility of publication bias, can be assessed for with the help of the Egger's test (48). However, applying this method is only appropriate when the numbers of effect sizes within the meta-analysis is >10 (62). Instead, we created a funnel plot and performed the Eggers' test (supplements) assessing all effect sizes used in this umbrella review (*k* = 38). It may serve as a rough assessment for overall-publication bias.

#### Sensitivity Analysis

Confounding factors (gender/sex, age, socioeconomic status) were in some meta-analyses adjusted for. The extracted values can be found in the (Supplementary Material Tables 1–3).

## Results

### Study Characteristics

**Figure 1:**
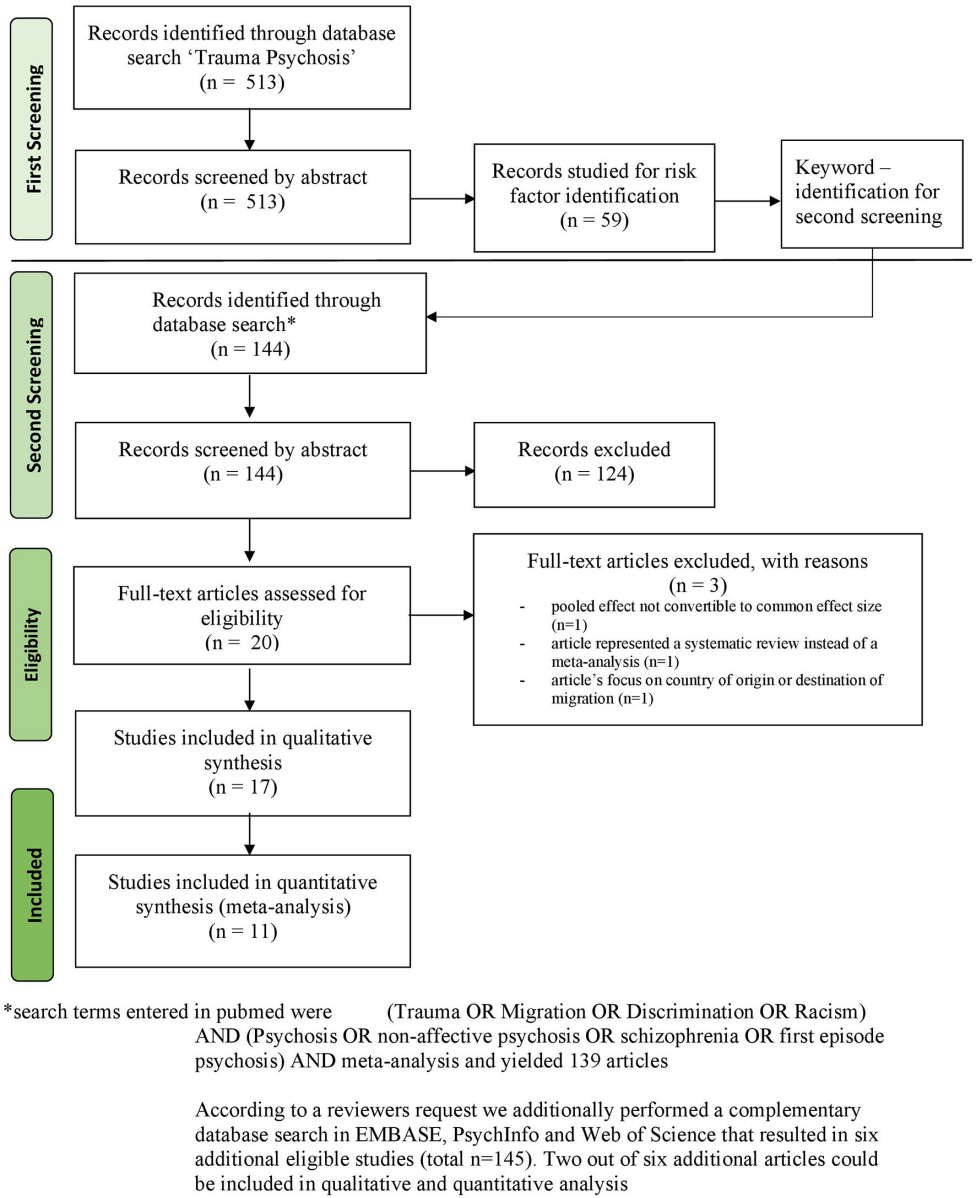
Flow diagram to summarize selection process, adapted from (40).

The flowchart in Figure 1 visualizes the search strategy for this study. The initially found 139 citations were reduced to 18 full-texts-assessed meta-analyses after application of the inclusion criteria. This number reduced further to 15 studies that could be included in the current umbrella review. The studies from Matheson et al. (63), Bonoldi et al. (19), and Nielssen et al. (64) had to be excluded due to the criteria mentioned above. The complementary search on Embase, PsychInfo, and Web of Science led to the inclusion of two other meta-analyses from Castillejos et al. (65) and Brandt et al. (9).

#### Quality of the Studies

A result of the AMSTAR-rating of 1–4 was considered low, 5–7 medium, and 8–11 of high quality, respectively. All included meta-analyses could be ranked as high quality except for Olbert et al. (66), Cannon et al. (45) and Castillejos et al. (65), which were estimated to be of medium quality. The meta-analysis of Bosqui et al. (6) made use of uncommon methods for weighting of the original studies, as the inverse of the quality score was used as a weighting factor for the calculation of summary effect sizes. However, our scoring still ranked this meta-analysis to be of high quality. Varese et al. (67) stated in their supplements that a quality rating for included original studies was not applicable, other authors (8) accounted for quality issues by reporting additional effect sizes that excluded papers with high risk of bias, which could, nevertheless, not be accounted for in the AMSTAR index. A table including the quality rating can be found in the (Supplementary Tables 1–3).

We were able to include effect sizes of 11 of the studies into one or more quantitative syntheses in form of meta-analyses (5, 8, 39, 66–70). Six further studies reported risk-factors that were exclusively described only in their study. As the calculation of a summary effect size requires at least two effect sizes from different meta-analyses, these studies could only be evaluated qualitatively (6, 16, 24, 36, 71–73).

Data for analysis were obtained from five articles for childhood adversities [Bailey et al. (16), de Sousa et al. (71); Pastore et al. (69); Rafiq et al. (70); Varese et al. (67)] covering the risk factors total childhood trauma, specific trauma types (sexual abuse, physical abuse, emotional abuse, neglect) and other childhood adversities as (bullying in childhood, parental death, and variations/impairments in parental communication). Some studies assessed the relation between certain psychotic symptoms (hallucination, delusions, dissociation, or positive and negative psychotic symptoms) (16, 70) and different kinds of trauma. Beards et al. (24) examined the association of psychosis to aversive adult life events. Data provided by Cannon et al. (36) covering obstetric complications were very detailed and can be found in the Supplementary Table 4.

Regarding migration, there are seven suitable meta-analyses that address different points [Anderson and Edwards (73), Bourque et al. (68), Cantor-Graae and Selten (5), Henssler et al. (8), Selten et al. (39), Castillejos et al. (65), Brandt et al. (9)]. They could be grouped according to whether they assessed differences in first and second generation migrants (5, 8, 39, 65, 68), a refugee status (9, 39) and effects associated to the age at migration (73).

Five studies examined the association of vulnerability for psychosis associated with ethnic discrimination proxied by skin color and ethnic density [Selten et al. (39), Cantor-Graae and Selten (5), Bourque et al. (68), Olbert et al. (66), Bosqui et al. (6)]. Two studies examined the relation between urbanicity and psychosis [Kirkbride et al. (72), Castillejos et al. (65)].

#### Quantitative and Qualitative Analysis

**Figure 2:**
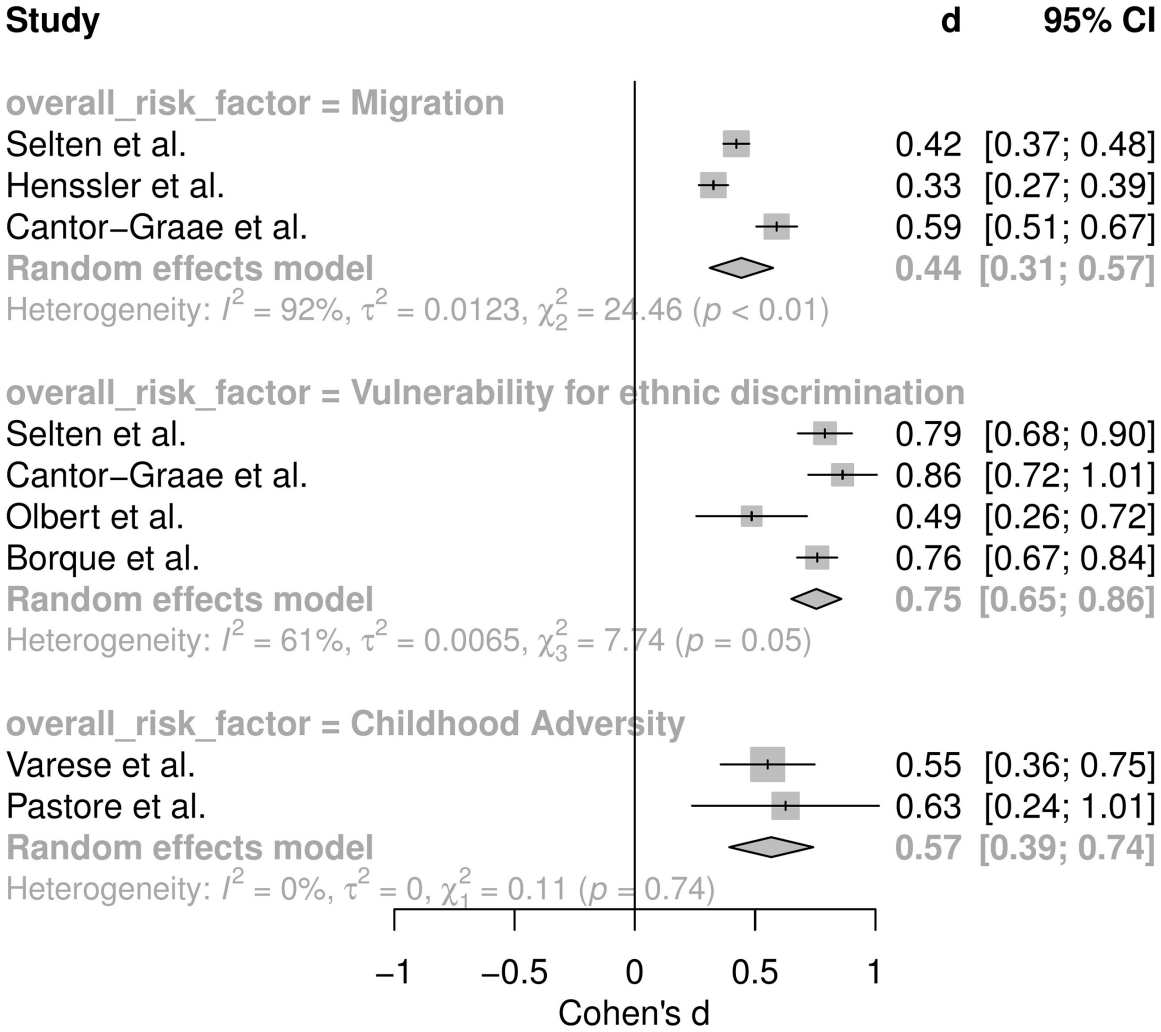
Meta-analysis comparing the summary effect sizes for migration, vulnerability for ethnic discrimination, and childhood adversity, respectively.

For the overall comparison (VI) between the summary effects, the following studies could be included for vulnerability for ethnic discrimination (5, 39, 66, 68), for childhood adversity (67, 69) and for migration (5, 8, 39), respectively. The Q-value for between group differences was significant *Q* = 13.77 (*p* = 0.001) for the overall comparison between the summary effects of (1) vulnerability for ethnic discrimination, (2) childhood trauma, and (3) migration, respectively. This means that the effects of vulnerability to discrimination, childhood trauma, and migration differ in their size concerning the risk for psychotic experiences: The pooled effect of vulnerability for ethnic discrimination was of medium size with high heterogeneity [*k* = 5; *d* = 0.77 (0.65–0.86); *p* < 0.001; *I*^2^ = 61.3%; *Q* = 7.74; τ^2^ = 0.75]. Heterogeneity in this context means that the results of the underlying meta-analyses vary highly. The summary size effect of childhood adversities also showed a medium effect size with low heterogeneity [*k* = 2; *d* = 0.57 (0.39–0.74); *p* < 0.001 *I*^2^ = 0%; *Q* = 0.11; τ^2^ = 0]. The summary size effect of risk factors related to migration was small with substantial heterogeneity [*k* = 2; *d* = 0.44 (0.31–0.57); *p* < 0.001; *I*^2^ = 91.8%; *Q* = 24.46, τ^2^ = 0.0123]. Reconversion of these values to facilitate interpretation yielded to OR = 3.90 (3.25–4.76), OR = 2.81 (2.03–3.83) and OR = 2.22 (1.75–2.81), respectively (Figure 2).

#### Childhood Adversities

Adjusted data for total childhood trauma did not differ much to the pooled effect (Supplementary Table 1A) with overlapping confidence intervals. Regarding specific symptoms, Bailey et al. (16) identified an association between childhood trauma and the severity of hallucinations and delusion, but not with the severity of negative symptoms (Supplementary Table 1A). Focusing on specific types of childhood trauma, emotional abuse displayed strongest associations to psychosis, followed by physical abuse, sexual abuse, and neglect (Ia) (Table 1). Bullying in childhood (IIb) as a type of childhood adversity resulted in a summary size effect of *k* = 2, *d* = 0.49 (0.37–0.62) which is slightly below the other forms of childhood traumatization, although with an overlapping confidence interval. Possible effects of parental death (Ic) displayed weaker summary effect sizes [*k* = 2; *d* = 0.12 (0.04–0.21)]. Variations/impairments in parental communication (Id) was examined only by one study (71) that identified very high effect sizes (Table 1) with medium heterogeneity. Aversive adult life events (Ie) were only assessed in one meta-analysis (24) that reported medium to high effect sizes of *d* = 0.64 (0.43–0.86) with high heterogeneity of *I*^2^ = 87.3%.

#### Migration

The pooled effect size of studies assessing the risk for first-generation migrants was higher than for those assessing for both generations, and those assessing for second-generation migrants only (IIa) (Table 2), but all confidence intervals overlapped and can be ranked as medium (60). Data with a high-quality rating were preferred in this analysis. The medium quality study displayed slightly higher effect sizes for risk and second generation migrants compared with high quality studies (Supplementary Table 2). The summary effect size for a refugee status was medium (IIb) (Table 2). Adjusted data for confounders age, sex/gender, and socioeconomic status displayed slightly lower effect sizes (Supplementary Table 2).

One study examined the effect of age of migration and the risk of psychosis (73), and found highest incidences among migrants aged 0–2 and 3–6 years when migrating, while incidences were comparable with the native age group for migrants who migrated at the age of 19–29 years (IIc) (Supplementary Table 2).

#### Vulnerability for Ethnic Discrimination

The association between vulnerability for racist discrimination and the risk of psychosis was strongest for migrants living in areas of low ethnic density (IIIb) [effect from one meta-analysis (6)]; *d* = 0.83 (0.45–1.19), and for individuals with minority status and a black skin color (IIIa) {summary effect [*k* = 5; *d* = 0.77 (0.67–0.87)]} (Table 3).

Urbanicity and the link to NAP and SCZ yielded in a large summary effect size (IV) (Table 4). Obstetric complications displayed varying effect sizes (V) but are only included in the supplement (Supplementary Table 4) due to quality issues and the extent of the study: diabetes in pregnancy showed highest impact of all risk factors examined [*d* = 1.12 (0.17–2.09)], followed by placental abruption, birth weight <2,000 g, emergency cesarean section, and congenital malformations.

#### Heterogeneity

Heterogeneity values of original meta-analyses was low (*I*^2^ <25%), medium (*I*^2^ <50%), high (*I*^2^ <75%), and substantial (*I*^2^ > 75%), respectively, for the following reported effect sizes in this study: low for total childhood trauma, sexual abuse, sexual abuse and severity of hallucinations, physical abuse, emotional abuse, neglect, neglect and severity of hallucinations, and severity of delusions, emotional and physical neglect and dissociation, bullying in childhood, parental death, minority position skin color, other high and low ethnic density; medium for childhood trauma and severity of hallucinations, childhood trauma and severity of delusions, and positive psychotic symptoms, sexual abuse and severity of delusions and positive psychotic symptoms, variations in parental communication; high for childhood trauma and dissociation and severity of negative psychotic symptoms, sexual abuse and severity of negative and positive psychotic symptoms, neglect and severity of negative psychotic symptoms, second-generation migrants, refugee status, age at migration (36, 7–12 years), majority position skin color white, minority position skin color black; and substantial for aversive adult life events, first- and second-generation migrants (high quality studies), urbanicity, first-generation migrants only, age at migration (0–2, 13–18, 19–29 years), all migration studies that adjusted for confounders (age, sex/gender/socioeconomic status). Values of heterogeneity were not available for childhood trauma studies that adjusted for confounders, first and second generation migrants (medium quality studies), and data on obstetric complications (Supplementary Tables 1–3).

#### Publication Bias

Within the data extracted for this study, there was slight evidence for publication bias in the article of Rafiq et al. (70) regarding childhood trauma and the risk for schizophrenia, of Pastore et al. (69) regarding childhood trauma and the risk for psychotic disorder, of Anderson et al. (73) regarding age at migration and the risk for psychotic disorder, of Olbert et al. (66) regarding black individuals and diagnosis of schizophrenia, and of De Sousa et al. (71) regarding variation in parental communication and the risk of psychotic disorder. In addition, there was considerable publication bias within data of Bailey et al. (16) regarding childhood trauma and severity of hallucinations (Supplementary Table 1A). The funnel plot created for the purpose of this study displayed some asymmetry and the Egger's test was significant (*p* = 0.007) suggesting publication bias (see Supplements).

## Discussion

The main findings of our umbrella review confirm the substantial increase in the risk to develop non-affective psychosis when exposed to trauma or discrimination. In fact, the strongest increase in this risk was associated with vulnerability for ethnic discrimination proxied by visible minority status (with high heterogeneity), while numerically lower effects were found for childhood adversities (with low heterogeneity) and migration (with substantial heterogeneity). In further analysis including all poolable and non-poolable effect sizes, most substantial effects were observed for exposure to variation/impairment of parental communication, small size of the local ethnic group of a member of that group (low ethnic density), black skin color, and emotional abuse, followed by aversive adult life events, physical abuse, urbanicity (with substantial heterogeneity), sexual abuse, bullying, neglect, refugee status and further factors associated with social isolation and discrimination, including an extraordinarily high ethnic density that may indicate social separation and marginalization. A low but still significant effect was found for parental death. Is it plausible that such diverse factors all contribute to the manifestation of schizophrenia and related psychotic disorders? A computational approach of psychotic disorders suggests that imprecise prior knowledge biases information processing toward sensory input, thus increasing errors of prediction and, hence, volatility of the representation of the environment (27, 74). We and others have suggested that imprecision of prior knowledge may be caused by both primarily biological (e.g., inflammation impairing neural information processing) as well as social factors, the latter including cultural differences and experiences of traumatization and discrimination (27). In this perspective, traumatization or discrimination may induce existential anxiety and evoke feelings of being threatened, not only in outright dangerous but already ambivalent or ambiguous social contexts (75). In such contexts, stress exposure can stimulate phasic dopamine release, which reduces all too complex or chaotic environmental input by attributing salience to certain environmental cues, thus increasing the signal to noise ratio (26, 75). However, salience may then also be attributed to otherwise irrelevant stimuli, which contributes to delusional mood and delusion (76). Finally, delusion formation, associated with higher order processing, may help to further reduce complexity and information overflow, however, at the expense of flexible belief adaptations (26, 27). Altogether, experiences of trauma, discrimination, and social exclusion can challenge prior knowledge and trust in social interactions, thus promoting a focus on environmental input, particularly when a person feels threatened, which stimulates a cascade of (partly compensatory) alterations in information processing that result in key symptoms of psychosis. While this model provides a plausible path to psychosis, it has to be emphasized that stress is known to have differing neuroplastic effects depending on age (77), so traumatizing and aversive events may have rather specific neurobiological effects in the development and clinical course of psychosis. Our findings are not suggestive to assume that either adult or childhood trauma exposure have a greater impact on the development of psychosis. Nevertheless, the here examined risk factors can be very aversive or are directly traumatic (2) and therefore, suggest that therapy of trauma should more regularly be available for persons with psychotic experiences.

These findings indicate a dire need for the therapy of trauma among persons with psychotic experiences. However, there is a substantial lack of evidence. As far as psychotherapeutic approaches are concerned, treatment of trauma and specifically post-traumatic stress disorder (PTSD) is based on a robust body of evidence favoring trauma-focused interventions that include exposure and/or cognitive restructuring as a central component (78–80). Trauma focused cognitive behavioral therapy (CBT), Prolonged Exposure (PE), Cognitive Processing Therapy (CPT), Eye Movement Desensitization and Reprocessing (EMDR), Brief Eclectic Psychotherapy (BEP), Narrative Exposure Therapy (NET), and written narrative exposure are therefore recommended by the national and international guidelines (81). Trauma-focused psychological therapies like EMDR and NET have been shown to be effective in improving symptoms for refugees and asylum seekers with PTSD (82). Compared with single-event PTSD, multicomponent and more flexible interventions were recommended for patients exposed to complex, war-related or childhood-onset trauma, who also suffer from disturbances of self-organization like emotional dysregulation (80, 83). However, evidence is less compelling regarding the treatment of PTBS in the presence of comorbid mental disorders, especially psychoses, as these usually represent exclusion criteria (84). Similarly, many studies support the efficacy of psychotherapies like CBT and family interventions in psychotic disorders (85–88), but evidence from randomized-controlled research on psychological interventions for PTSD in patients with severe mental illness is still scarce. A recent Cochrane review and meta-analysis identified only four eligible trials (89). In a seminal study, van den Berg et al. compared EMDR, PE, and a waiting group in (*n* = 155) patients with a lifetime diagnosis of psychosis or mood disorder with psychotic features. Patients who received one of the active therapies achieved greater reductions of PTSD symptoms and significantly more often lost PTSD diagnosis than those in the waiting list group. Results were stable at 6–12 months of follow-up (90). Mueser et al. compared two RCTs of mixed patient groups with severe mental disorders 16 sessions of a CBT for PTSD program with standard or brief treatments and reported small to medium improvements of PTSD symptoms in the intervention groups at 6 months (91, 92). A study focusing patients with schizophrenia and exhibiting post-traumatic stress symptoms (*n* = 61) found no effect of a 16-session cognitive restructuring intervention compared with standard care (93). In another smaller study (*n* = 50) patients with schizophrenia, bipolar or not otherwise specified psychoses with a documented history of childhood trauma were administered either 10 group sessions of Acceptance and Commitment Therapy (ACT) or treatment as usual. Results indicated improvements in brief psychiatric rating scale (BPRS), anxiety, and emotional acceptance, but not in trauma-related symptoms in the ACT sample (94). However, in contrast to previous concerns of worsening psychotic symptoms by exposure to trauma-associated material (95), no adverse events were reported by any RCT and controlled safety studies (95, 96). Meanwhile, a number of theoretical approaches target the risk of developing psychosis conferred by interpersonal trauma or aim at the treatment of comorbid post-traumatic symptoms (97–99). Moreover, mentalization-based psychotherapy was shown to improve functional outcome in psychotic patients (100) and may, like other psychodynamic approaches focusing reflective functioning, attachment, and interpersonal regulation (101–103) be complemented by trauma-specific treatment components, at least in integrative and team-based settings.

Adjunctive non-psychotherapeutic approaches could address both trauma and psychotic experiences but remain poorly researched. As a lack of social support and discrimination as well as social exclusion (6, 8) contribute to psychosis and traumatization, therapeutic group-sessions may support a sense of belonging for these patients. When direct verbal interaction becomes difficult, non-verbal treatment strategies such as occupational or art therapies have a long-standing role in facilitating engagement and affiliation (104).

Creative therapies are recommended as therapeutic offers for all patients with psychosis or schizophrenia by the National Institute for Health and Clinical Excellence (105) (NICE) and may be specifically useful for the alleviation of negative symptoms. Regarding their effectiveness, there is inconclusive evidence for the treatment of psychosis: in a recent meta-analysis, Law and Convey (106) investigated the effects of different kinds of art therapy (arts, music, dance, and body-orientated psychotherapy), by analyzing nine RCTs. They concluded that in contrast to the NICE endorsement, there is a lack of evidence for any reduction in total or positive symptoms of schizophrenia. Significant reductions of negative symptoms in favor of art therapy provided in groups have been reported, but this effect was not stable in trials using blind assessment of outcomes only. A previous review conducted by (104) included qualitative and quantitative research methods focusing on art therapy (not including dance, music or other approaches) for persons with psychotic disorders. They analyzed two high quality RCTs (107–109) and other quantitative studies with conflicting results. Five high-quality qualitative articles suggested that clients and therapists considered art therapy as beneficial and meaningful (104).

As language and cultural differences can present challenges in the treatment of traumatized adults, art therapies may also be helpful to facilitate communication and support social contact and engagement. Due to a weak evidence base (few studies with methodological limitations, heterogeneity of studies), there are so far no recommendations for non-verbal approaches in psychiatric guidelines for the treatment of persons with trauma (including the APA guideline, 2017 for the treatment of PTSD in adults, or the NICE guideline, 2018 for PTSD). As a lacking sense of belonging seems to play an important role within the formation of traumatization, NICE guidelines [2018] recommend peer groups, which should be instructed by therapeutic professionals. Art may facilitate such groups as a treatment option in a non-pathologizing manner. Schouten et al. (110) reported some evidence that art therapy interventions are effective in reducing trauma symptom severity and anxiety: three out of six controlled studies included in their systematic review reported a significant decrease of depression in individuals with PTSD. In a more recent systematic review Baker et al. (111) also included music and drama therapy but found low to very low evidence for each therapy form.

Altogether, to improve treatment options for individuals with psychosis and traumatization, future research could focus on individual experiences and assess outcome measurements including social functioning, well-being, mentalization, and self-efficacy (104).

### Strengths and Limitations

The robustness of umbrella reviews depends on the robustness and comparability of underlying meta-analyses, which themselves depend on the robustness and comparability of original studies. Our umbrella review suggests that risk factors including overall childhood trauma are influenced by publication bias of various degrees, and summary effect sizes might therefore be overestimated. High and substantial heterogeneity was found within most risk factors in the field of migration and vulnerability for ethnic discrimination proxied by skin-color. This study examined both diagnosed traumatic events and potentially traumatizing events. This heterogeneity of potentially traumatizing factors may limit the generalization of our findings; however, it emphasizes the relevance of severely aversive events that could potentially be prevented by targeted interventions. Based on the reviewed meta-analyses, we grouped social risk factors potentially associated with trauma. This umbrella review is limited by the fact that the examined constructs, childhood adversities' “vulnerability for ethnic discrimination and migration” are based on our own classification of the literature; however, previous work on migration (8, 39), evidence from longitudinal and retrospective studies for vulnerability for ethnic discrimination (68, 112), and studies on childhood trauma (16, 67), including a possible dose response relation, suggest that these are highly relevant factors. A limitation of our approach is that pathways to psychosis, thus, addressed may vary considerably, because neurobiological correlates of trauma differ considerably between childhood and adulthood (77). Also, we do not address other potentially relevant traumatic experiences during adulthood independent of migration and minority status due to a lack of meta-analyses: we only found one meta-analysis of Beards et al. who examined adult life events and grouped these potentially traumatizing events in adulthood together (24). Further research should also reassess the influence of regional characteristics that might influence the effect. Furthermore, heterogeneity can also be caused by diagnostic or sample bias, as studies including patients with schizophrenia only were pooled together with a result from samples including a broader definition of non-affective psychotic disorders or patient groups that included psychotic experiences or psychotic symptoms. Although we did our best to avoid overlap reporting by excluding certain summary effect sizes to ensure independent underlying samples for a new pooled summary effect size, we cannot be fully certain that all overlaps could be identified correctly. We, thus, applied a random effect model for the calculation of summary effect sizes assuming a distribution of true summary effect sizes accounting for each sample, respectively.

Finally, a quantitative assessment of confounders was not possible in this study due to limited data. Results of individual meta-analyses suggest that age, sex/gender, and socioeconomic status confound the data for first- and second-generation migration (Supplementary Table 2), and lower values would be found after an adjustment.

## Conclusions

Our umbrella review strongly suggests that in addition to childhood trauma, social exclusion, racist discrimination based on skin color and minority status, as well as other forms of adult traumatization and adversity substantially contribute to the risk of psychosis. In spite of these rather strong effect sizes, there is only limited evidence for interventions using psychotherapy, art therapy, or other non-psychotherapeutic approaches that address both trauma and psychotic experiences. Future studies need to address how the effects of diverse severely aversive and traumatizing experiences of patients with psychosis may best be treated. This includes psychosocial interactions focusing on the community in a case of systematic social exclusion, as well as psychotherapeutic interventions aiming at specific traumatizing experiences (113, 114). Given the strong effect of indicators of social and racist exclusion (6, 10) on psychosis risk, interventions at a societal level could include fighting stigma and racism, and providing social support to reduce poverty and marginalization, and to increase solidarity and community inclusion (35, 115, 116).

## Data Availability Statement

The code and datasets generated for this study can be found on Github: https://github.com/lveaor/Umbrella-review-2021.

## Author Contributions

LV, JK, CM, YT, and AH planned and prespecified the study protocol and wrote and conceived the paper. LV, JK, and AH screened the literature, and extracted and checked the relevant data. LV and JK did the statistical analysis. All authors fulfilled the ICMJE Criteria for Authorship (117).

## Funding

This study is supported, in part, by CRC-TR 265. JK is supported by the Charité Clinician-Scientist Program of the Berlin Institute of Health. (https://orcid.org/0000-0001-8155-3683).

## Conflict of Interest

The authors declare that the research was conducted in the absence of any commercial or financial relationships that could be construed as a potential conflict of interest.

## Publisher's Note

All claims expressed in this article are solely those of the authors and do not necessarily represent those of their affiliated organizations, or those of the publisher, the editors and the reviewers. Any product that may be evaluated in this article, or claim that may be made by its manufacturer, is not guaranteed or endorsed by the publisher.
