# Supplementary material for: Traumatic Events, Social Adversity and Discrimination as Risk Factors for Psychosis - An Umbrella Review
Source: Front Psychiatry. 2021 Oct 22;12:665957. doi: 10.3389/fpsyt.2021.665957 (PMC8569921; doi:10.3389/fpsyt.2021.665957)
Supplement: Supplementary file 1 [file Table_1.docx]

| **Table S1a** Childhood trauma, other childhood adversities and psychosis risk  Specific trauma types, adjusted data, severity of symptoms depending on trauma type  Summary effects and qualitative review of included meta-analyses | | | | | | | | | | | |  |  |
| --- | --- | --- | --- | --- | --- | --- | --- | --- | --- | --- | --- | --- | --- |
| **Study** |  | Year of publication | Dia-gnosis | k | n1 | n2 | Statistical value and variance | | **Summary statistical value^+^** or common size effect^++^ and variance | I^2^ | Q | Risk of publication bias, test applied | AMSTAR quality rating |
| **Childhood trauma** | | | | | | | | |  |  |  |  |  |
| **Total childhood trauma** | | | | | | | | | | | |  |  |
| Varese et al. |  | 2012 | PD, PS | 18 | 2078 | 1980 | | OR = 2.72 (1.91–3.88) | d = 0.55 (0.36–0.75) |  |  | n, ^1, 3^ | 8/11 |
| Pastore et al. |  | 2020 | PD | 5 |  |  | | OR = 3.12 (1.54–6.3) ^***^ | d = 0.62 (0.24–1.01) |  |  | y,^1,3,4^ | 10/11 |
| **total** |  |  |  |  |  |  | |  | **d = 0.57 (0.39**–**0.74)** | 0 | 0.11^ns^ |  |  |
| **Specific trauma type** | | | | | | | | | | | |  |  |
| **Sexual abuse** | | | | | | | | | | | |  |  |
| Rafiq et al. |  | 2018 | SCZ, Dis | 13 |  |  | | r = 0.30 (0.18–0.41) ^****^ | d = 0.63 (0.37–0.90) | 68.09 | 37.6 | n,^1^ y,^3^ | 10/11 |
| Varese et al. |  | 2012 | PD, PS | 20 | >4105 | >9871 | | OR=2.38(1.98–2.87) ^****^ | d = 0.48 (0.38–0.58) | 44.9 | 34.5^**^ | n, ^1, 3^ | 8/11 |
| **Total** |  |  |  |  |  |  | |  | **d = 0.50 (0.39**–**0.62)** | 10.0 | 1.11^*^ |  |  |
| **Physical abuse** | | | | | | | | | | | |  |  |
| Rafiq et al. |  | 2018 | SCZ, Dis | 13 |  |  | | r = 0.32 (0.24–0.40) ^****^ | d = 0.68 (0.49–0.87) | 37.4 | 19.18 | n,^1^ | 10/11 |
| Varese et al. |  | 2012 | PD, PS | 13 | > 945 | > 2678 | | OR=2.95(2.25–3.88) ^****^ | d = 0.58 (0.45–0.75) | 74.9 | 47.8^****^ | n, ^1, 3^ | 8/11 |
| **total** |  |  |  |  |  |  | |  | **d = 0.63 (0.51**–**0.74)** | 0 | 0.49^ns^ |  |  |
| **Emotional abuse** | | | | | | | | | | | |  |  |
| Rafiq et al. |  | 2018 | SCZ, Dis | 12 |  |  | | r = 0.41 (0.27–0.54) ^****^ | d = 0.92 (0.56–1.28) | 76.7 | 47.11 | n,^1^ | 10/11 |
| Varese et al. |  | 2012 | PD, PS | 6 | 914 | 6597 | | OR=3.40(2.06–5.62) ^****^ | d = 0.68 (0.40–0.95) | 78.3 | 23.1^****^ | n, ^1, 3^ | 8/11 |
| **total** |  |  |  |  |  |  | |  | **d = 0.77 (0.53**–**1.01)** | 11.6 | 1.13^*^ |  |  |
| **Neglect** | | | | | | | | | | | |  |  |
| Rafiq et al. |  | 2018 | SCZ, Dis | 23 |  |  | | r = 0.22 (0.15–0.28) ^****^ | d = 0.44 (0.30–0.58) | 36.5 | 34.6^****^ | n,^1^ | 10/11 |
| Varese et al. |  | 2012 | PD, PS | 7 | > 802 | > 958 | | OR=2.90(1.71–4.92) ^****^ | d = 0.58 (0.30–0.88) | 81.8 | 32.9^**^ | n, ^1, 3^ | 8/11 |
| **total** |  |  |  |  |  |  | |  | **d = 0.47 (0.34**–**0.60)** | 0 | 0.76^ns^ |  |  |
| **Childhood trauma – adjusted data** | | | | | | | | | | | | | |
| **sex/gender** | | | | | | | | | | | |  |  |
| Varese et al. |  | 2012 | PD, PS | 10 |  |  | | OR = 2 .52 (2.00 - 3.19) | d = 0.51 (0.38 - 0.64) |  |  | n, ^1, 3^ | 8/11 |
| **age** | | | | | | | | | | | | | |
| Varese et al. |  | 2012 | PD, PS | 9 |  |  | | OR = 2.57 (2.00 - 3.31) | d = 0.52 (0.38 - 0.66) |  |  | n, ^1, 3^ | 8/11 |
| **socioeconomic status** | | | | | | | | | | | | | |
| Varese et al. |  | 2012 | PD, PS | 6 |  |  | | OR = 3.01 (1.98 - 4.58) | d = 0.61 (0.38 - 0.84) |  |  | n, ^1, 3^ | 8/11 |
| **sex/gender + age + socioeconomic status adjustment** | | | | | | | | | | | | |  |
| Varese et al. |  | 2012 | PD, PS | 12 |  |  | | OR = 2.72 (2.08 - 3.68) | d = 0.55 (0.40 - 0.72) |  |  | n, ^1, 3^ | 8/11 |
| \| **Childhood trauma, severity of symptoms** \|  \|  \| \| --- \| --- \| --- \| | | | | | | | | | | | | | |
| Rafiq et al. |  | 2018 | SCZ, Dis | 20 |  |  | | r = 0.39 (0.31 - 0.46) ^****^ | d = 0.85 (0.65 - 1.04) | 64.4 | 50.6 | n,^1^ | 8/11 |
| Bailey et al. |  | 2018 | PD, sH | 12 |  |  | | r = 0.12 (0.12 - 0.28) ^****^ | d = 0.41 (0.24 - 0.57) | 42.5 | 19.1^*^ | y,^1,5^ | 10/11 |
| Bailey et al. |  | 2018 | PD, sDel | 10 |  |  | | r = 0.17 (0.08 - 0.26) ^****^ | d = 0.35 (0.16 - 0.54) | 48.8 | 17.6^**^ | n, ^1^ | 10/11 |
| Bailey et al. |  | 2018 | PD, sPPS | 18 |  |  | | r = 0.14 (0.09 - 0.19) ^****^ | d = 0.29 (0.18 - 0.39) | 40.4 | 28.5^**^ |  | 10/11 |
| Bailey et al. |  | 2018 | PD,sNPS | 15 |  |  | | r = 0.05 (-0.01 - 0.11)* | d = 0.10 (-0.02 - 0.21) | 51.1 | 29.2^***^ | n, ^1^ | 10/11 |
| **Sexual abuse** | | | | | | | | | | | |  |  |
| Bailey et al. |  | 2018 | PD, sH | 8 |  |  | | r = 0.24 (0.16 - 0.31)^****^ | d = 0.49 (0.32 - 0.66) | 0 | 6.6 | n, ^1^ | 10/11 |
| Bailey et al. |  | 2018 | PD, sDel | 7 |  |  | | r = 0.14 (0.02 - 0.26)^**^ | d = 0.28 (0.04 - 0.53) | 42.0 | 10.4^*^ | n, ^1^ | 10/11 |
| Bailey et al. |  | 2018 | PD, sPPS | 10 |  |  | | r = 0.20 (0.11 - 0.29)^****^ | d = 0.41 (0.22 - 0.61) | 40.1 | 15.0^*^ |  | 10/11 |
| Bailey et al. |  | 2018 | PD,sNPS | 9 |  |  | | r = 0.09 (0.03 - 0.21)^*^ | d = 0.18 (0.06 - 0.43) | 57.0 | 18.6^**^ | n, ^1^ | 10/11 |
| **Physical neglect** | | | | | | | | | | | |  |  |
| Rafiq et al. |  | 2018 | SCZ, Dis | 11 |  |  | | r = 0.30 (0.23 - 0.37)^****^ | d = 0.63 (0.47 - 0.80) | 0 | 9.5 | n, ^1^ | 8/11 |
| **Emotional neglect** | | | | | | | | | | | | | |
| Rafiq et al. |  | 2018 | SCZ, Dis | 11 |  |  | | r = 0.11 (0.02 - 0.20)^**^ | d = 0.22 (0.04 - 0.41) | 20.5 | 12.6 | n, ^1^ | 8/11 |
| **Neglect** | | | | | | | | | | | |  |  |
| Bailey et al. |  | 2018 | PD, sH | 5 |  |  | | r = 0.14 (0.02 - 0.25)^**^ | d = 0.28 (0.04 - 0.51) | 2.8 | 4.1 | n, ^1^ | 10/11 |
| Bailey et al. |  | 2018 | PD, sDel | 5 |  |  | | r = 0.06 (0.05 - 0.17)^*^ | d = 0.12 (0.10 - 0.35) | 0 | 3.1 | n, ^1^ | 10/11 |
| Bailey et al. |  | 2018 | PD, sPPS | 5 |  |  | | r = 0.13 (0.02 - 0.27)^*^ | d = 0.26 (0.04 - 0.55) | 63.1 | 10.8^**^ |  | 10/11 |
| Bailey et al. |  | 2018 | PD,sNPS | 8 |  |  | | r = 0.14 (0.04 - 0.24)^***^ | d = 0.29 (0.09 - 0.49) | 61.6 | 18.2^**^ | n, ^1^ | 10/11 |
| **Other childhood adversities** | | | | | | | | | | | |  |  |
| **Bullying in childhood** | | | | | | | | | | | |  |  |
| Pastore et al. |  | 2020 | PD | 8 |  |  | | OR =2.28(1.64–4.34) | d = 0.54 (0.27–0.81) | 92.0 | 87.0^****^ | n, ^1,3^ | 10/11 |
| Varese et al. |  | 2012 | PD, PS | 6 |  |  | | OR =2.39(1.83–3.11) ^****^ | d = 0.48 (0.33–0.63) | 73.9 | 19.1^***^ | n, ^1, 3^ | 8/11 |
| **total** |  |  |  |  |  |  | |  | **d = 0.49 (0.37**–**0.62)** | 0 | 0.16^ns^ |  |  |
| **Parental death** | | | | | | | | | | | |  |  |
| Pastore et al. |  | 2020 | PD | 4 |  |  | | OR = 1.24(1.06–1.44) | d = 0.12 (0.03–0.20) | 0.0 | 3.1 | n, ^1,3^ | 10/11 |
| Varese et al. |  | 2012 | PD, PS | 8 | > 1347 | > 1326 | | OR = 1.70(0.82–3.53) ^*^ | d = 0.29 (-0.11–0.70) | 80.2 | 35.4^****^ | n, ^1,3^ | 8/11 |
| **total** |  |  |  |  |  |  | |  | **d = 0.12 (0.04**–**0.21)** | 0 | 0.71^ns^ |  |  |
| **Variations/impairments in parental communication** | | | | | | | | | | | |  |  |
| De Sousa et al. |  | 2014 | PD | 19 | 864 | 847 | | g = 0.97 (0.76-1.18) ^****^ | d = 0.97 (0.76-1.18) | 46.5 | 33.4^****^ | y, ^1,3,^n,^4^ | 8/11 |
| PD = psychotic disorder; NAP = non-affective psychosis. SCZ = schizophrenia; PS = psychotic symptoms; Dis = dissociation  k = number of effect sizes; n1 = number of cases; n2 = number of controls,  OR = Odds ratio; r = Pearson’s correlation coefficient; g = Hedge’s g; d = Cohen’s d; **^+^** = pooled result of several meta-analyses written in bold letters ;^++^ = one meta analysis available, result converted in common effect size and presented qualitatively; Q = Cochran’s Q heterogeneity statistics; I^2^= I^2^-index for heterogeneity; ^*^ = p < 0.3; ^**^ = p < 0.05; ^***^ = p < 0.01;^****^ = p < 0.001;  ^1^Eggers test(1),^2^visual inspection of the funnel plot for asymmetry, ^3^trim and fill method (2), ^4^ Begg and Mazumdars rank correlation method (3), ^5^ Fail-save-N-method (4) n = no, y = yes | | | | | | | | | | | |  |  |

| **Table S1b** Adult life events and psychosis risk  Qualitative review of included meta-analysis | | | | | | | | | | |  | | |  | | |  |
| --- | --- | --- | --- | --- | --- | --- | --- | --- | --- | --- | --- | --- | --- | --- | --- | --- | --- |
| **Study** | Year of publication | Dia-gnosis | k | n1 | n2 | Statistical value and variance | | Common size effect^++^ and variance | I^2^ | Q | | Risk of publication bias | AMSTAR quality rating | | |  |  |
| **Adult life events** | | | | | | | | | | |  | | |  | | |  |
| Beards et al. | 2013 | PD, PE | 13 | 2218 | 19389 | | OR = 3.19 (2.15–4.75) | d = 0.64 (0.42–0.86) | 87.3 |  | |  | | | 8/11 | | |
| \| PD = psychotic disorder; PE = psychotic experience; k = number of effect sizes; n1 = number of cases; n2 = number of controls; OR = odds ratio. d = Cohen’s d; ^++^ = one meta analysis available, result converted in common effect size and presented qualitatively; Q = Cochran’s Q heterogeneity statistics; I^2^= I^2^-index for heterogeneity; \| \| --- \| | | | | | | | | | | | | | | | | | |
|  | | | | | | | | | | | | | | | | |  |

| **Table S1c** Urbanicity and psychosis risk  Summary effects and qualitative review of included meta-analyses | | | | | | | | | | | | | | | | | | |  |
| --- | --- | --- | --- | --- | --- | --- | --- | --- | --- | --- | --- | --- | --- | --- | --- | --- | --- | --- | --- |
| **Study** | Year of publication | Dia-gnosis | k | n1 | n2 | Statistical value and variance | | **Summary statistical value^+^** or common size effect^++^ and variance | | I^2^ | | Q | | Risk of publication bias | | AMSTAR quality rating | | | |
| **Urbanicity** | | | | | | | | |  | |  | |  | |  | |  | |  |
| Kirkbride et al. | 2012 | NAP | 9 |  |  | | IRR=1.02 (1.02–1.03) ^***^ | d = 0.01 (0.01–0.02) | |  | |  | |  | | 10/11 | |  |  |
| Kirkbride et al. | 2012 | SCZ | 15 |  |  | | IRR=1.03 (1.01–1.03) ^***^ | d = 0.02 (0.01–0.02) | |  | |  | |  | | 10/11 | |  |  |
| Castillejos et el. | 2018 | NAP | 5 |  |  | | IRR=2.25 (2.00-2.52) ^****^ | d= 0.45 (0.38–0.51) | |  | |  | |  | | 7/11 | |  |  |
| Castillejos et el. | 2018 | SCZ | 3 |  |  | | IRR=1.64 (1.38-1.95) ^***^ | d= 0.27 (0.18-0.37) | |  | |  | |  | | 7/11 | |  |  |
| **total** |  |  |  |  |  | |  | **d= 0.57 (0.39-0.74)** | | 98.5 | | 206.71^***^ | |  | |  | |  |  |
| \| NAP = non-affective psychosis. SCZ = schizophrenia; k = number of effect sizes; n1 = number of cases; n2 = number of controls; OR = Odds ratio; d = Cohen’s d; **^+^** = pooled result of several meta-analyses written in bold letters ;^++^ = one meta analysis available, result converted in common effect size and presented qualitatively; Q = Cochran’s Q heterogeneity statistics; I^2^= I^2^-index for heterogeneity; ^*^ = p < 0.3; ^**^ = p < 0.05; ^***^ = p < 0.01;^****^ = p < 0.001; \| \| --- \| | | | | | | | | | | | | | | | | | | |  |

| **Table S2** First and Second generation migrants, refugee status and psychosis risk  Adjusted data  Summary effects and qualitative review of included meta-analyses | | | | | | | | | | | | | |  |
| --- | --- | --- | --- | --- | --- | --- | --- | --- | --- | --- | --- | --- | --- | --- |
| **Study** |  | Year of publication | Dia-gnosis | k | n1 | n2 | Statistical value and variance | | **Summary statistical value^+^** or common size effect^++^ and variance | I^2^ | Q | Risk of publication bias | AMSTAR quality rating |  |
| **First and second generation migrants, high quality studies** | | | | | | | | | | | |  |  |  |
| Selten et al. |  | 2020 | NAP | 15 | 4896 | 18040 | | RR = 2.15 (1.95–2.37)^*^ | d = 0.42 (0.37–0.48) | 94.7 |  | n,^1^ | 9/11 |  |
| Henssler et al. |  | 2020 | NAP | 25 |  |  | | RR = 1.81(1.62–2.02) | d = 0.33 (0.27–0.39) | 97.6 |  | n,^1,2^ | 8/11 |  |
| Cantor-Gr. et al. |  | 2005 | SCZ | 50 | 3092 | 27130 | | RR = 2.9 (2.5–3.4) | d = 0.59 (0.51–0.67) |  | 68.3^**^ | n, ^2^ | 8/11 |  |
| **total** |  |  |  |  |  |  | |  | **d = 0.44 (0.31–0.57)** | 91.8 | 24.5^****^ |  |  |  |
| **First and second generation migrants, medium quality studies** | | | | | | | | | | | |  |  |  |
| Castillejos et el. |  | 2018 | NAP | 6 |  |  | | IRR = 3.08(2.62–3.63) ^***^ | d = 0.62 (0.53–0.71) |  |  |  | 7/11 |  |
| Castillejos et el. |  | 2018 | SCZ | 4 |  |  | | IRR = 2.74(2.04–3.67) ^***^ | d = 0.56 (0.39–0.72) |  |  |  | 7/11 |  |
| **First generation migrants** | | | | | | | | | | | | | |  |
| Selten et al.. |  | 2020 | PD | 29 | 14351 | 84701 | | RR = 2.55 (2.31–2.82) | d = 0.52 (0.46–0.57) | 97.9 |  | n,^1^ | 9/11 |  |
| Henssler et al. |  | 2020 | NAP | 20 |  |  | | RR = 1.81(1.59–2.07) | d = 0.33 (0.26–0.40) | 97.6 |  | n,^1,2^ | 8/11 |  |
| Cantor-Gr. et al. |  | 2005 | SCZ | 40 | 2846 | 26785 | | RR = 2.7 (2.3– 3.2) | d = 0.55 (0.46–0.64) |  | 55.4^**^ | n, ^2^ | 8/11 |  |
| Bourque et al.. |  | 2011 | PD | 61 | 5556 | 33160 | | IRR = 2.3 (2.0– 2.7) | d = 0.46 (0.38–0.55) | 94.4 | 1071.0^***^ | n, ^2^ | 10/11 |  |
| **total** |  |  |  |  |  |  | |  | **d = 0.46 (0.37**–**0.56)** | 85.2 | 20.3^****^ |  |  |  |
| **Second generation migrants** | | | | | | | | | | | | | |  |
| Selten et al.. |  | 2020 | PD | 13 |  |  | | RR =1.78 (1.66– 1.90)^***^ | d = 0.32 (0.28–0.35) | 94.2 |  | n,^1^ | 9/11 |  |
| Henssler et al. |  | 2020 | NAP | 13 |  |  | | RR = 1.82(1.66–1.99) | d = 0.33 (0.28–0.38) | 90.5 |  | n,^1,2^ | 8/11 |  |
| Cantor-Gr. et al. |  | 2005 | SCZ | 7 | 474 | 8895 | | RR = 4.5 (1.5–13.1) | d = 0.82 (0.22–1.42) | 4.5 | 55.4 | n, ^2^ | 8/11 |  |
| Bourque et al.. |  | 2011 | PD | 28 | 4515 | 24360 | | IRR = 2.1 (1.8– 2.5) | d = 0.41 (0.32–0.51) | 91.1 | 303.0^***^ | n, ^2^ | 10/11 |  |
| **total** |  |  |  |  |  |  | |  | **d = 0.34 (0.29- 0.40)** | 53.4 | 6.4^**^ |  |  |  |
| **First and second-generation migration, adjusted data** | | | | | | | | | | | | | |  |
| **Age** | | | | | | | | | | | | | |  |
| Selten et al.. |  | 2020 | PD | 8 | 4342 | 12135 | | RR = 1.72 (1.57 - 1.87) | d = 0.30 (0.25 – 0.35) | 98.0 |  |  | 9/11 |  |
| Henssler et al. |  | 2020 | NAP | 25 |  |  | | RR = 1.78 (1.62 – 1.95) | d = 0.32 (0.27 – 0.37) | 96.7 |  |  | 8/11 |  |
| **Age + sex/ gender** | | | | | | | | | | | | | |  |
| Selten et al. |  | 2020 | NAP | 8 |  |  | | RR = 1.74 (1.59 - 1.90) | d = 0.31 (0.26 - 0.35) | 98.0 |  |  | 9/11 |  |
| **Age + sex/ gender + socioeconomic status** | | | | | | | | | | | | | |  |
| Selten et al. |  | 2020 | PD | 10 |  |  | | RR = 1.53 (1.41 - 1.67) | d = 0.23 (0.19 - 0.28) | 95.2 |  |  | 9/11 |  |
| Selten et al. |  | 2020 | NAP | 8 |  |  | | RR = 1.55 (1.42 - 1.69) | d = 0.24 (0.19 - 0.29) | 95.3 |  |  | 9/11 |  |
| **Age at migration** |  |  |  |  |  |  | |  |  |  |  |  |  |  |
| **0-2 years** |  |  |  |  |  |  | |  |  |  |  |  |  |  |
| Anderson et al. |  | 2020 | PD | 5 |  |  | | IRR=1.85(1.39- 2.47)^***^ | d = 0.34 (0.18 - 0.50) | 75.5 |  | y,^1^ | 8/11 |  |
| **3-6 years** |  |  |  |  |  |  | |  |  |  |  |  |  |  |
| Anderson et al. |  | 2020 | PD | 5 |  |  | | IRR=1.85(1.56 - 2.20)^**^ | d = 0.34 (0.25 - 0.43) | 63.5 |  | y,^1^ | 8/11 |  |
| **7-12 years** |  |  |  |  |  |  | |  |  |  |  |  |  |  |
| Anderson et al. |  | 2020 | PD | 5 |  |  | | IRR=1.73(1.17 - 2.37)^***^ | d = 0.30 (0.09 - 0.48) | 60.3 |  | y,^1^ | 8/11 |  |
| **13-18 years** |  |  |  |  |  |  | |  |  |  |  |  |  |  |
| Anderson et al. |  | 2020 | PD | 5 |  |  | | IRR=0.93(0.60- 1.40)^****^ | d =-0.04 (-0.28 - 0.19) | 94.8 |  | y,^1^ | 8/11 |  |
| **19-29 years** |  |  |  |  |  |  | |  |  |  |  |  |  |  |
| Anderson et al. |  | 2020 | PD | 5 |  |  | | IRR= 0.93(0.60-1.44)^****^ | d =-0.04 (-0.28 - 0.20) | 97.6 |  | y,^1^ | 8/11 |  |
| **Refugees** |  |  |  |  |  |  | |  |  |  |  |  |  |  |
| Brandt et al. |  | 2019 | NAP | 10 |  |  | | RR =2.52 (1.78–3.57)^****^ | d = 0.51 (0.32-0.70) | 98.0 |  | n,^1^ |  |  |
| Selten et al. |  | 2020 | NAP | 4 |  |  | | RR= 1.88(1.57-2.24) | d=0.35(0.25-0.45) | 91.4 |  |  |  |  |
| **total** |  |  |  |  |  |  | |  | **d= 0.41(0.25-0.56)** | 54.6 | 2.20^*^ |  |  |  |
| PD = psychotic disorder; NAP = non-affective psychosis. SCZ = schizophrenia; k = number of effect sizes; n1 = number of cases; n2 = number of controls RR = risk ratio. IRR = incidence rate ratio. d = Cohen’s d; **^+^** = pooled result of several meta-analyses written in bold letters ;^++^ = one meta analysis available, result converted in common effect size and presented qualitatively Q = Cochran’s Q heterogeneity statistics; I^2^= I^2^-index for heterogeneity; ^*^ = p < 0.3; ^**^ = p < 0.05; ^***^ = p < 0.01;^****^ = p < 0.001  ^1^Eggers test(1),^2^visual inspection of the funnel plot for asymmetry; n = no; y = yes | | | | | | | | | | | | | |  |

| **Table S3** Vulnerability for racist discrimination and psychosis risk  Summary effects and qualitative review of included meta-analyses | | | | | | | | | | | | | | | | | | | | |  |
| --- | --- | --- | --- | --- | --- | --- | --- | --- | --- | --- | --- | --- | --- | --- | --- | --- | --- | --- | --- | --- | --- |
| **Study** | Year of publication | F/S | Dia-gnosis | | k | | n1 | | n2 | | Statistical value and variance | **Summary statistical value^+^** or common size effect^++^ and variance | I^2^ | Q | Risk of publication bias | | AMSTAR quality rating | | |  |  |
| **Majority position/skin-color white** | | | | | | | | | | | | | | | | | | | | |  |
| Selten et al. | 2020 | F+S | NAP | | 19 | |  | |  | RR = 1.65 (1.46–1.85) | | d = 0.27 (0.21–0.34) | 97.1 |  | |  | | 9/11 | | |  |
| Cantor-Gr. et al. | 2005 | F+S | SCZ | 16 | | 799 | | 15902 | | RR = 2.3 (1.8–3.0) | | d = 0.46 (0.32–0.61) |  |  | | n, ^2^ | | 8/11 | | |  |
| Bourque et al. | 2011 | F | PD | | 19 | | 1808 | | 20853 | IRR = 1.8 (1.6–2.1) | | d = 0.33 (0.26–0.41) | 89.7 | 175.4^***^ | | n, ^2^ | | 10/11 | | |  |
| Bourque et al. | 2011 | F | PD | | 4 | | 243 | | 5566 | IRR = 1.9 (1.2–3.0) | | d = 0.35 (0.10–0.61) | 87.2 | 23.5^***^ | | n, ^2^ | | 10/11 | | |  |
| **total** |  |  |  | |  | |  | |  |  | | **d = 0.34 (0.26**–**0.41)** | 51.5 | 6.18 | |  | |  | | |  |
| **Minority position/skin-color black** | | | | | | | | | | | | | | | | | | |  |  |  |
| Selten et al. | 2020 | F+S | NAP | | 23 | |  | |  | RR =4.19(3.42–5.14) ^****^ | | d = 0.79 (0.68–0.90) | 94.3 |  | |  | | 9/11 | | |  |
| Cantor-Gr. et al. | 2005 | F+S | SCZ | | 16 | | 896 | | 24931 | RR = 4.8 (3.7–6.2) | | d = 0.86 (0.72–1.01) |  |  | | n, ^2^ | | 8/11 | | |  |
| Olbert et al. | 2018 | BI | SCZ | | 52 | | 863293 | | 2532655 | OR =2.42(1.59–3.66) ^****^ | | d = 0.49 (0.25–0.71) | 98.3 |  | | n,^1^y,^6^ | | 7/11 | | |  |
| Bourque et al. | 2011 | F | PD | | 18 | | 1711 | | 25255 | IRR = 4.0 (3.4–4.6) | | d = 0.76 (0.67–0.84) | 79 | 80.8^***^ | | n, ^2^ | | 10/11 | | |  |
| Bourque et al. | 2011 | F | PD | | 7 | | 127 | | 279 | IRR = 5.4 (3.2–8.8) | | d = 0.92 (0.64–1.19) | 78.9 | 28.4^***^ | | n, ^2^ | | 10/11 | | |  |
| **total** |  |  |  | |  | |  | |  |  | | **d = 0.77 (0.67–0.87)** | 54.8 | 8.85^**^ | |  | |  | | |  |
| **Minority position/skin-color other** | | | | | | | | | |  | |  |  |  | |  | |  | | |  |
| Selten et al. | 2020 | F+S | NAP | | 11 | |  | |  | RR = 1.73(1.41–2.14) | | d = 0.30 (0.18–0.42) | 95.1 |  | |  | | 9/11 | | |  |
| Cantor-Gr. et al. | 2005 | F+S | SCZ | | 11 | | 649 | | 13782 | RR = 2.2 (1.6–3.0) | | d = 0.43 (0.26–0.61) |  |  | | n, ^2^ | | 8/11 | | |  |
| Bourque et al. | 2011 | F | PD | | 16 | | 505 | | 14765 | IRR = 2.0 (1.6–2.5) | | d = 0.38 (0.26–0.51) | 84.7 | 97.8^***^ | | n, ^2^ | | 10/11 | | |  |
| Bourque et al. | 2011 | F | PD | | 5 | | 51 | | 8843 | IRR = 2.0 (1.0–4.0) | | d = 0.38 (0.00–0.76) | 73.8 | 15.3^***^ | | n, ^2^ | | 10/11 | | |  |
| **total** |  |  |  | |  | |  | |  |  | | **d = 0.36 (0.28**–**0.43)** | 0 | 1.7 | |  | |  | | |  |
| **Ethnic density** | | | | | | | | | | | | | | | | | | |  |  |  |
| **High** |  |  |  | |  | |  | |  |  | |  |  |  | |  | |  | | |  |
| Bosqoui et al. | 2014 |  | PD | | 5 | |  | |  | IRR = 2.52 (1.28–5.32) | | d = 0.51 (0.14–0.92) | 0 |  | | n,^5^ | | 9/11 | | |  |
| **Low** |  |  |  | |  | |  | |  |  | |  |  |  | |  | |  | | |  |
| Bosqoui et al. | 2014 |  | PD | | 5 | |  | |  | IRR=4.51(2.25–8.58) | | d = 0.83 (0.45–1.19) | 0 |  | | n,^5^ | | 9/11 | | |  |
| F = first generation migrant; S = second generation migrant; BI = black individuals; PD = psychotic disorder; NAP = non-affective psychosis. SCZ = schizophrenia; k = number of effect sizes; n1 = number of cases; n2 = number of controls OR = odds ratio. RR = risk ratio. IRR = incidence rate ratio. d = Cohen’s d; **^+^** = pooled result of several meta-analyses written in bold letters ;^++^ = one meta analysis available, result converted in common effect size and presented qualitatively; Q = Cochran’s Q heterogeneity statistics; I^2^= I^2^-index for heterogeneity ^*^ = p < 0.3; ^**^ = p < 0.05; ^***^ = p < 0.01;^****^ = p < 0.001;  ^1^Eggers test(1),^2^visual inspection of the funnel plot for asymmetry, ^3^trim and fill method (2), ^4^ Begg and Mazumdars rank correlation method (3), ^5^ Fail-save-N-method (4), ^6^Luis – Furuya – Kanamori (LFK) – index (5); n = no; y = yes | | | | | | | | | | | | | | | | | | | | |  |

| **Table S4** Obstetic complications and psychosis risk  Qualitative review of included meta-analyses | | | | | | | | | | | |
| --- | --- | --- | --- | --- | --- | --- | --- | --- | --- | --- | --- |
| **Factor** | Dia-gnosis | k | n1 | n2 | Statistical value and variance | | Common size effect and variance^++^ | I^2^ | Q | Risk of publication bias | AMSTAR quality rating |
| **Obstetic complications – all effect sizes from Cannon et al. 2002** | | | | | | | | | |  |  |
| Diabetes in pregnancy | SCZ | 2 |  |  | | OR = 7.6 (1.37 - 43.9)^**^ | d = 1.12 (0.17 - 2.09) |  |  | n,^2^ | 6/11 |
| Placental abruption | SCZ | 2 |  |  | | OR = 4.04 (0.89- 18.12)^*^ | d = 0.77 (-0.06 - 1.60) |  |  | n,^2^ | 6/11 |
| birth weight <2000g | SCZ | 2 |  |  | | OR =3.89(1.4 - 10.84)^***^ | d = 0.75 (0.19 - 1.31) |  |  | n,^2^ | 6/11 |
| emergency caesarean section | SCZ | 3 |  |  | | OR = 3.24 (1.4 - 7.5)^***^ | d = 0.65 (0.19 - 1.11) |  |  | n,^2^ | 6/11 |
| congenital malformations | SCZ | 3 |  |  | | OR = 2.35 (1.21 - 4.57)^**^ | d = 0.47 (0.11 - 0.84) |  |  | n,^2^ | 6/11 |
| uterine atony | SCZ | 2 |  |  | | OR= 2.29 (1.51 - 3.5)^****^ | d = 0.46 (0.23 - 0.69) |  |  | n,^2^ | 6/11 |
| rhesus variables (includes incompatibility, rhesus- negative mother, rhesus antibodies) | SCZ | 3 |  |  | | OR = 2.00 (1.01 - 3.96)^**^ | d = 0.38 (0.01 - 0.76) |  |  | n,^2^ | 6/11 |
| threatened premature delivery | SCZ | 2 |  |  | | OR = 1.98 (0.79 - 4.9)^**^ | d = 0.38 (-0.13 - 0.88) |  |  | n,^2^ | 6/11 |
| asphyxia | SCZ | 3 |  |  | | OR =1.74(1.15 - 2.62)^***^ | d = 0.31 (0.08 - 0.53) |  |  | n,^2^ | 6/11 |
| bleeding in pregnancy | SCZ | 6 |  |  | | OR = 1.69 (1.14-2.52)^***^ | d = 0.29 (0.07 - 0.51) |  |  | n,^2^ | 6/11 |
| birth weight <2500g | SCZ | 5 |  |  | | OR = 1.67(1.22- 2.29)^***^ | d = 0.28 (0.11 - 0.46) |  |  | n,^2^ | 6/11 |
| head circumference < 32cm | SCZ | 2 |  |  | | OR = 1.38 (0.97 - 1.91)^*^ | d = 0.18 (-0.02 - 0.36) |  |  | n,^2^ | 6/11 |
| smoking in pregnancy | SCZ | 2 |  |  | | OR = 1.38(0.88- 2.14)^*^ | d = 0.18 (-0.07 - 0.42) |  |  | n,^2^ | 6/11 |
| preeclampsia | SCZ | 6 |  |  | | OR = 1.36 (0.99 - 1.85)^**^ | d = 0.17 (-0.01 - 0.34) |  |  | n,^2^ | 6/11 |
| anaemia in pregnancy | SCZ | 3 |  |  | | OR = 1.26 (0.69 - 2.28)^**^ | d = 0.13 (-0.20 - 0.45) |  |  | n,^2^ | 6/11 |
| gestational age < 37 weeks | SCZ | 5 |  |  | | OR = 1.22 (0.9 - 1.65)^*^ | d = 0.11 (-0.06 - 0.28) |  |  | n,^2^ | 6/11 |
| small for gestational age | SCZ | 5 |  |  | | OR = 1.21 (0.91 - 1.61)^*^ | d = 0.11 (-0.05 - 0.26) |  |  | n,^2^ | 6/11 |
| induction of labour | SCZ | 4 |  |  | | OR = 1.18(0.89 - 1.56)^*^ | d = 0.09 (-0.06 - 0.25) |  |  | n,^2^ | 6/11 |
| gestational age >42 weeks | SCZ | 3 |  |  | | OR = 1.08 (0.69 - 1.68) | d = 0.04 (-0.20 - 0.29) |  |  | n,^2^ | 6/11 |
| child stayed in hospital after mother discharged | SCZ | 3 |  |  | | OR = 1.07 (0.79 - 1.44) | d = 0.04 (-0.13 - 0.20) |  |  | n,^2^ | 6/11 |
| forceps delivery of vacuum extraction | SCZ | 7 |  |  | | OR = 1.07 (0.85 - 1.35) | d = 0.04 (-0.09 - 0.17) |  |  | n,^2^ | 6/11 |
| birth length < 49 cm | SCZ | 3 |  |  | | OR = 1.06 (0.86 - 1.31) | d = 0.03 (-0.08 - 0.15) |  |  | n,^2^ | 6/11 |
| cephalopelvic disproportion | SCZ | 2 |  |  | | OR = 1.04 (0.28 - 3.82) | d = 0.02 (-0.70 - 0.74) |  |  | n,^2^ | 6/11 |
| cord around neck | SCZ | 2 |  |  | | OR = 1.03 (0.81 - 1.31) | d = 0.02 (-0.12 - 0.15) |  |  | n,^2^ | 6/11 |
| caesarean section | SCZ | 5 |  |  | | OR = 0.99 (0.7 - 1.41) | d = -0.01(-0.20 - 0.19) |  |  | n,^2^ | 6/11 |
| birth weight < 2500g and premature | SCZ | 4 |  |  | | OR = 0.96 (0.62 - 1.46) | d = -0.02(-0.26 - 0.21) |  |  | n,^2^ | 6/11 |
| nonvertex presentation | SCZ | 6 |  |  | | OR = 0.89 (0.67 - 1.2) | d = -0.06(-0.22 - 0.10) |  |  | n,^2^ | 6/11 |
| breech delivery | SCZ | 3 |  |  | | OR = 0.87 (0.38 - 1.97) | d = -0.08(-0.53 - 0.37) |  |  | n,^2^ | 6/11 |
| urinary tract infection in pregnancy | SCZ | 3 |  |  | | OR = 0.86 (0.48 - 1.55) | d = -0.08(-0.40 - 0.24) |  |  | n,^2^ | 6/11 |
| nonspontaneous delivery | SCZ | 2 |  |  | | OR = 0.63 (0.39 - 1.01)^*^ | d = -0.25(-0.52 - 0.01) |  |  | n,^2^ | 6/11 |
| SCZ = schizophrenia; k = number of effect sizes; n1 = number of cases; n2 = number of controls, OR = odds ratio, d = Cohen’s d; ^++^ = one meta analysis available, result converted in common effect size and presented qualitatively; Q = Cochran’s Q heterogeneity statistics; I^2^= I^2^-index for heterogeneity ^*^ = p < 0.3; ^**^ = p < 0.05; ^***^ = p < 0.01;^****^ = p < 0.001; ^2^visual inspection of the funnel plot for asymmetry; n = no | | | | | | | | | | | |

1. Egger M, Davey Smith G, Schneider M, Minder C. Bias in meta-analysis detected by a simple, graphical test. BMJ (Clinical research ed). 1997;315(7109):629-34.

2. Duval S, Tweedie R. Trim and fill: A simple funnel-plot-based method of testing and adjusting for publication bias in meta-analysis. Biometrics. 2000;56(2):455-63.

3. Begg CB, Mazumdar M. Operating characteristics of a rank correlation test for publication bias. Biometrics. 1994;50(4):1088-101.

4. Rosenthal R. The file drawer problem and tolerance for null results. Psychological bulletin. 1979;86(3):638-41.

5. Furuya-Kanamori L, Barendregt JJ, Doi SAR. A new improved graphical and quantitative method for detecting bias in meta-analysis. Int J Evid Based Healthc. 2018;16(4):195-203.
