# Supplementary material for: Traumatic Events, Social Adversity and Discrimination as Risk Factors for Psychosis - An Umbrella Review
Source: Front Psychiatry. 2021 Oct 22;12:665957. doi: 10.3389/fpsyt.2021.665957 (PMC8569921; doi:10.3389/fpsyt.2021.665957)
Supplement: Supplementary file 2 [file Table_2.docx]

Publication bias

| **Author** | **Funnel plot (1)** | **Eggers test (2)** | **Fail-Safe N test (3)** | **LFK index (4)** | **Duval S Tweedie**  **Trim and Fill (5)** | **Begg and Mazumdars rank correlation method (6)** | **Information not provided** |
| --- | --- | --- | --- | --- | --- | --- | --- |
| **Selten et al. 2020 (7)** | Modest evidence of PB for risk of any PD |  |  |  |  |  |  |
| **Henssler et al. 2020 (8)** | No evidence for PB | p=0.643  no evidence for PB |  |  |  |  |  |
| **Rafiq et al. 2018 (9)** |  | CT, SMI P = 0.012 for  CT, SCZ; CT, PersD not significant? |  |  | CT, SMI: 7 hypothetical studies included  CT, SCZ : 4 missing studies  CT, PersD: 2 missing |  |  |
| **Varese et al. 2012 (10)** |  | Egger’s test not significant for all analyses |  |  | In aggregated analyses (not included in this umbrella review)  9 missing studies  Patient-control studies  7 missing studies,  CCS, Prospect. studies  No missing effect |  |  |
| **Bailey et al.**  **2018 (11)** |  | Publication bias for CT and sH, not for SA/N and sH, not for CT/SA/N and sD/ sNPS; no report for PPS | For CT and sH  fail safe N = 119 |  |  |  |  |
| **Cantor-Graae et al. 2005 (12)** | No sign for PB in any data-set used |  |  |  |  |  |  |
| **Anderson et al. 2020 (13)** | Some asymmetry |  |  |  |  |  |  |
| **Beards et al. 2013 (14)** |  |  |  |  |  |  | x |
| **Olbert et al. 2018 (15)** | Minor assymetry | No evidence for publication bias |  | -1.7 |  |  |  |
| **Bosqui et al. 2014 (16)** |  |  | For low ethnic density 127  For high ethnic density 36 |  |  |  |  |
| **Bourque et al. 2011 (17)** | Generation-specific no asymmetry |  |  |  |  |  |  |
| **Kirkbride et al 2012 (18)** |  | Black Caribbean: p=0.7 – no evidence for PB |  |  |  |  |  |
| **Pastore et al. 2020 (19)** |  | For CT significant  No PB for PaD, B |  |  | For CT 5 studies trimmed  No PB for PaD, B | For CT significant  No PB for PaD, B |  |
| **De Sousa et al.2014 (20)** |  | Suggests some PB  Intercept at 1.49 (-0.54 – 3.52), t = 1.55 p=0.7 |  |  | 2 potential missing but recomputed point estimate not likely to be affected | No PB |  |
| **Cannon et al. 2002 (21)** | No evidence for PB |  |  |  |  |  |  |
| **Castillejos et al. 2018 (22)** |  |  |  |  |  |  | x |
| **Brandt et al.**  **2019** |  | No evidence for PB |  |  |  |  |  |

CT = childhood trauma, PaD = Parental Death, B = bullying, SA = sexual abuse, N = neglect, sH = severity of Hallucinations, sD = severity of delusions, sPPS = severity of positive psychotic symptoms, sNPS = severity of negative psychotic symptoms, CCS = case control studies, SMI = severe mental illness, SCZ = schizophrenia PB = publication bias, PersD = Personality disorder, PD = Psychotic disorder

1. Egger M, Smith GD, Schneider M, Minder C. Bias in meta-analysis detected by a simple, graphical test. BMJ. 1997;315(7109):629-34.

2. Egger M, Davey Smith G, Schneider M, Minder C. Bias in meta-analysis detected by a simple, graphical test. BMJ (Clinical research ed). 1997;315(7109):629-34.

3. Rosenthal R. The file drawer problem and tolerance for null results. Psychological bulletin. 1979;86(3):638-41.

4. Furuya-Kanamori L, Barendregt JJ, Doi SAR. A new improved graphical and quantitative method for detecting bias in meta-analysis. Int J Evid Based Healthc. 2018;16(4):195-203.

5. Duval S, Tweedie R. Trim and fill: A simple funnel-plot-based method of testing and adjusting for publication bias in meta-analysis. Biometrics. 2000;56(2):455-63.

6. Begg CB, Mazumdar M. Operating characteristics of a rank correlation test for publication bias. Biometrics. 1994;50(4):1088-101.

7. Selten JP, van der Ven E, Termorshuizen F. Migration and psychosis: a meta-analysis of incidence studies. Psychol Med. 2020;50(2):303-13.

8. Henssler J, Brandt L, Müller M, Liu S, Montag C, Sterzer P, et al. Migration and schizophrenia: meta-analysis and explanatory framework. Eur Arch Psychiatry Clin Neurosci. 2020;270(3):325-35.

9. Rafiq S, Campodonico C, Varese F. The relationship between childhood adversities and dissociation in severe mental illness: a meta-analytic review. Acta Psychiatr Scand. 2018;138(6):509-25.

10. Varese F, Smeets F, Drukker M, Lieverse R, Lataster T, Viechtbauer W, et al. Childhood adversities increase the risk of psychosis: a meta-analysis of patient-control, prospective- and cross-sectional cohort studies. Schizophr Bull. 2012;38(4):661-71.

11. Bailey T, Alvarez-Jimenez M, Garcia-Sanchez AM, Hulbert C, Barlow E, Bendall S. Childhood Trauma Is Associated With Severity of Hallucinations and Delusions in Psychotic Disorders: A Systematic Review and Meta-Analysis. Schizophr Bull. 2018;44(5):1111-22.

12. Cantor-Graae E, Selten JP. Schizophrenia and migration: a meta-analysis and review. Am J Psychiatry. 2005;162(1):12-24.

13. Anderson KK, Edwards J. Age at migration and the risk of psychotic disorders: a systematic review and meta-analysis. Acta Psychiatr Scand. 2020;141(5):410-20.

14. Beards S, Gayer-Anderson C, Borges S, Dewey ME, Fisher HL, Morgan C. Life events and psychosis: a review and meta-analysis. Schizophr Bull. 2013;39(4):740-7.

15. Olbert CM, Nagendra A, Buck B. Meta-analysis of Black vs. White racial disparity in schizophrenia diagnosis in the United States: Do structured assessments attenuate racial disparities? J Abnorm Psychol. 2018;127(1):104-15.

16. Bosqui TJ, Hoy K, Shannon C. A systematic review and meta-analysis of the ethnic density effect in psychotic disorders. Soc Psychiatry Psychiatr Epidemiol. 2014;49(4):519-29.

17. Bourque F, van der Ven E, Malla A. A meta-analysis of the risk for psychotic disorders among first- and second-generation immigrants. Psychol Med. 2011;41(5):897-910.

18. Kirkbride JB, Errazuriz A, Croudace TJ, Morgan C, Jackson D, Boydell J, et al. Incidence of schizophrenia and other psychoses in England, 1950-2009: a systematic review and meta-analyses. PLoS One. 2012;7(3):e31660.

19. Pastore A, de Girolamo G, Tafuri S, Tomasicchio A, Margari F. Traumatic experiences in childhood and adolescence: a meta-analysis of prospective studies assessing risk for psychosis. Eur Child Adolesc Psychiatry. 2020.

20. de Sousa P, Varese F, Sellwood W, Bentall RP. Parental communication and psychosis: a meta-analysis. Schizophr Bull. 2014;40(4):756-68.

21. Cannon M, Jones PB, Murray RM. Obstetric complications and schizophrenia: historical and meta-analytic review. Am J Psychiatry. 2002;159(7):1080-92.

22. Castillejos MC, Martín-Pérez C, Moreno-Küstner B. Incidence of psychotic disorders and its association with methodological issues. A systematic review and meta-analyses. Schizophr Res. 2019;204:458-9.
