# Supplementary material for: Traumatic Events, Social Adversity and Discrimination as Risk Factors for Psychosis - An Umbrella Review
Source: Front Psychiatry. 2021 Oct 22;12:665957. doi: 10.3389/fpsyt.2021.665957 (PMC8569921; doi:10.3389/fpsyt.2021.665957)
Supplement: Supplementary file 3 [file Table_3.DOCX]

Quality assessment:

AMSTAR index (2007)(1)

| Author | a priori design? | Duplicate study selection & data extraction? | Performance of comprehensive literature search? | Status of publication used as inclusion criteria? | List of studies included and excluded provided? | Characteristics of included studies provided? | Scientific quality of included studies assessed, documented? | Scientific quality of included studies used appropriately for formulating conclusions? | Methods used to combine studies appropriate? | Assessment of likelihood of publication bias? | Conflict of interest stated? | AMSTAR rating |
| --- | --- | --- | --- | --- | --- | --- | --- | --- | --- | --- | --- | --- |
| Selten et al. 2020 (2) | y | y | y | y (published peer review only) | n | y | y | y | y | y | y | 9/11 |
| Henssler et al. 2020 (3) | y | y | y | n | n | y | n | y | y | y | y | 8/11 |
| Rafiq et al. 2018 (4) | y | y | y | y + unpublished | n | y | y | y | y | y | y | 10/11 |
| Varese et al. 2012 (5) | y | y | y | y + unpublished | n | y | n | n | y | y | y | 8/11 |
| Bailey et al.  2018 (6) | y | y | y | y + unpublished | n | y | y | y | y | y | y | 10/11 |
| Cantor-Graae et al. 2005 (7) | y | y | y | y (published peer review only) | n | y | n | y | y | y | n | 8/11 |
| Anderson et al. 2020 (8) | y | y | y | y (published peer-review only | n | y | n | n | y | y | y | 8/11 |
| Beards et al. 2013 (9) | y | n | y | y (published peer review only) | n | y | y | y | y | n | y | 8/11 |
| Olbert et al. 2018 (10) | y | y | y | y (published peer review only) | n | y | n | n | y | y | n | 7/11 |
| Bosqui et al. 2014 (11) | y | y | y | y (published peer review only) | n | y | y | y | n, quality scores used as weighting factor | y | y | 9/11 |
| Bourque et al. 2011 (12) | y! | y | y | y (published peer review only) | n | y | y | y | y | y | y | 10/11 |
| Kirkbride et al 2012 (13) | y | y | y | y + unpublished | n | y | y | y | y | y | y | 10/11 |
| Pastore et al. 2020 (14) | y | y | y | y (published peer review only) | n | y | y | y | y | y | y | 10/11 |
| De Sousa et al.2014 (15) | n | y | y | y + unpublished | n | y | n | y | y | y | y | 8/11 |
| Cannon et al. 2002 (16) | y! | n | y | n | n | y | n | n | y | y | n | 6/11 |
| Castillejos et al. 2018 (17) | n | y | y | y | n | y | y | y | y | n | n | 7/11 |
| Brandt et al. 2019 (18) | y | y | y | y (published only) | n | y | y | y | y | y | y | 9/11 |

1. Shea BJ, Grimshaw JM, Wells GA, Boers M, Andersson N, Hamel C, et al. Development of AMSTAR: a measurement tool to assess the methodological quality of systematic reviews. BMC Medical Research Methodology. 2007;7(1):10.

2. Selten JP, van der Ven E, Termorshuizen F. Migration and psychosis: a meta-analysis of incidence studies. Psychol Med. 2020;50(2):303-13.

3. Henssler J, Brandt L, Müller M, Liu S, Montag C, Sterzer P, et al. Migration and schizophrenia: meta-analysis and explanatory framework. Eur Arch Psychiatry Clin Neurosci. 2020;270(3):325-35.

4. Rafiq S, Campodonico C, Varese F. The relationship between childhood adversities and dissociation in severe mental illness: a meta-analytic review. Acta Psychiatr Scand. 2018;138(6):509-25.

5. Varese F, Smeets F, Drukker M, Lieverse R, Lataster T, Viechtbauer W, et al. Childhood adversities increase the risk of psychosis: a meta-analysis of patient-control, prospective- and cross-sectional cohort studies. Schizophr Bull. 2012;38(4):661-71.

6. Bailey T, Alvarez-Jimenez M, Garcia-Sanchez AM, Hulbert C, Barlow E, Bendall S. Childhood Trauma Is Associated With Severity of Hallucinations and Delusions in Psychotic Disorders: A Systematic Review and Meta-Analysis. Schizophr Bull. 2018;44(5):1111-22.

7. Cantor-Graae E, Selten JP. Schizophrenia and migration: a meta-analysis and review. Am J Psychiatry. 2005;162(1):12-24.

8. Anderson KK, Edwards J. Age at migration and the risk of psychotic disorders: a systematic review and meta-analysis. Acta Psychiatr Scand. 2020;141(5):410-20.

9. Beards S, Gayer-Anderson C, Borges S, Dewey ME, Fisher HL, Morgan C. Life events and psychosis: a review and meta-analysis. Schizophr Bull. 2013;39(4):740-7.

10. Olbert CM, Nagendra A, Buck B. Meta-analysis of Black vs. White racial disparity in schizophrenia diagnosis in the United States: Do structured assessments attenuate racial disparities? J Abnorm Psychol. 2018;127(1):104-15.

11. Bosqui TJ, Hoy K, Shannon C. A systematic review and meta-analysis of the ethnic density effect in psychotic disorders. Soc Psychiatry Psychiatr Epidemiol. 2014;49(4):519-29.

12. Bourque F, van der Ven E, Malla A. A meta-analysis of the risk for psychotic disorders among first- and second-generation immigrants. Psychol Med. 2011;41(5):897-910.

13. Kirkbride JB, Errazuriz A, Croudace TJ, Morgan C, Jackson D, Boydell J, et al. Incidence of schizophrenia and other psychoses in England, 1950-2009: a systematic review and meta-analyses. PLoS One. 2012;7(3):e31660.

14. Pastore A, de Girolamo G, Tafuri S, Tomasicchio A, Margari F. Traumatic experiences in childhood and adolescence: a meta-analysis of prospective studies assessing risk for psychosis. Eur Child Adolesc Psychiatry. 2020.

15. de Sousa P, Varese F, Sellwood W, Bentall RP. Parental communication and psychosis: a meta-analysis. Schizophr Bull. 2014;40(4):756-68.

16. Cannon M, Jones PB, Murray RM. Obstetric complications and schizophrenia: historical and meta-analytic review. Am J Psychiatry. 2002;159(7):1080-92.

17. Castillejos MC, Martín-Pérez C, Moreno-Küstner B. Incidence of psychotic disorders and its association with methodological issues. A systematic review and meta-analyses. Schizophr Res. 2019;204:458-9.

18. Brandt L, Henssler J, Müller M, Wall S, Gabel D, Heinz A. Risk of Psychosis Among Refugees: A Systematic Review and Meta-analysis. JAMA Psychiatry. 2019;76(11):1133-40.
