# Supplementary figures and images for: Traumatic Events, Social Adversity and Discrimination as Risk Factors for Psychosis - An Umbrella Review
Source: Front Psychiatry. 2021 Oct 22;12:665957. doi: 10.3389/fpsyt.2021.665957 (PMC8569921; doi:10.3389/fpsyt.2021.665957)

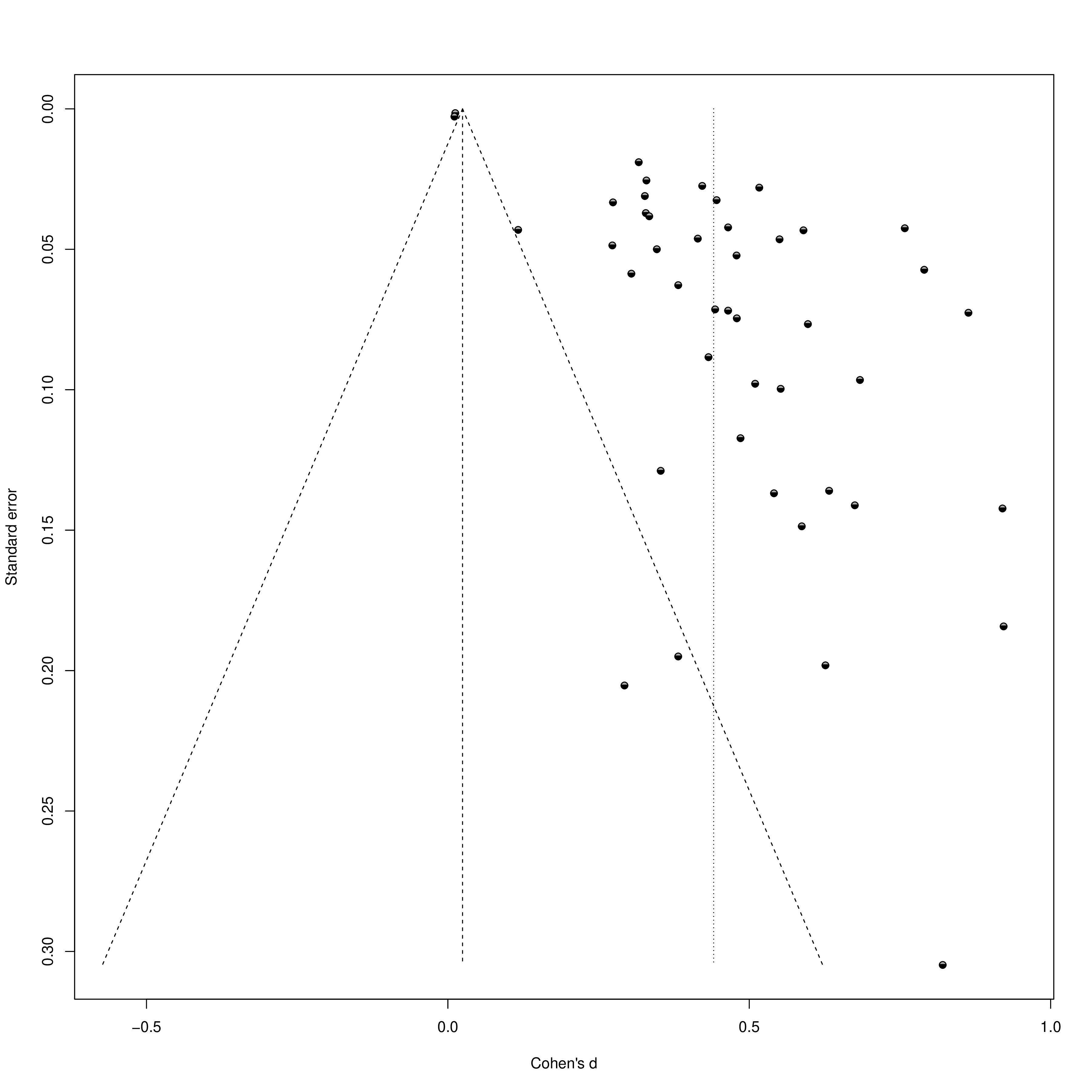

Supplement: Supplementary file 4 [file Image_1.jpg]
